# Commensal Bacteria-Induced Inflammasome Activation in Mouse and Human Macrophages Is Dependent on Potassium Efflux but Does Not Require Phagocytosis or Bacterial Viability

**DOI:** 10.1371/journal.pone.0160937

**Published:** 2016-08-09

**Authors:** Kejie Chen, Nanda Kumar N. Shanmugam, Michael A. Pazos, Bryan P. Hurley, Bobby J. Cherayil

**Affiliations:** 1 Mucosal Immunology and Biology Research Center, Department of Pediatrics, Massachusetts General Hospital, and Harvard Medical School, Boston, Massachusetts, United States of America; 2 College of Veterinary Medicine, Sichuan Agricultural University, Ya’an, Sichuan, China; Virginia Polytechnic Institute and State University, UNITED STATES

## Abstract

Gut commensal bacteria contribute to the pathogenesis of inflammatory bowel disease, in part by activating the inflammasome and inducing secretion of interleukin-1ß (IL-1ß). Although much has been learned about inflammasome activation by bacterial pathogens, little is known about how commensals carry out this process. Accordingly, we investigated the mechanism of inflammasome activation by representative commensal bacteria, the Gram-positive *Bifidobacterium longum* subspecies *infantis* and the Gram-negative *Bacteroides fragilis*. *B*. *infantis* and *B*. *fragilis* induced IL-1ß secretion by primary mouse bone marrow-derived macrophages after overnight incubation. IL-1ß secretion also occurred in response to heat-killed bacteria and was only partly reduced when phagocytosis was inhibited with cytochalasin D. Similar results were obtained with a wild-type immortalized mouse macrophage cell line but neither *B*. *infantis* nor *B*. *fragilis* induced IL-1ß secretion in a mouse macrophage line lacking the nucleotide-binding/leucine-rich repeat pyrin domain containing 3 (NLRP3) inflammasome. IL-1ß secretion in response to *B*. *infantis* and *B*. *fragilis* was significantly reduced when the wild-type macrophage line was treated with inhibitors of potassium efflux, either increased extracellular potassium concentrations or the channel blocker ruthenium red. Both live and heat-killed *B*. *infantis* and *B*. *fragilis* also induced IL-1ß secretion by human macrophages (differentiated THP-1 cells or primary monocyte-derived macrophages) after 4 hours of infection, and the secretion was inhibited by raised extracellular potassium and ruthenium red but not by cytochalasin D. Taken together, our findings indicate that the commensal bacteria *B*. *infantis* and *B*. *fragilis* activate the NLRP3 inflammasome in both mouse and human macrophages by a mechanism that involves potassium efflux and that does not require bacterial viability or phagocytosis.

## Introduction

The inflammasome is a cytosolic, multi-subunit protein complex that is involved in the processing and secretion of the pro-inflammatory cytokine interleukin-1ß (IL-1ß). It is assembled in response to a number of exogenous and endogenous stimuli associated with infection or tissue damage [1,2]. Such stimuli are usually sensed by a specific member of the nucleotide-binding/leucine-rich repeat (NLR) family of proteins and trigger the formation of the inflammasome oligomer, which consists of the NLR, the inactive cysteine-aspartate protease pro-caspase 1 and, often, the adaptor ASC (apoptosis-associated speck-like protein with a caspase recruitment domain). Assembly of this complex leads to the proximity-induced autoproteolytic activation of pro-caspase 1. Active caspase 1 cleaves pro-IL-1ß to convert it to the mature form that is competent for secretion. IL-1ß secretion usually occurs in response to 2 distinct signals–signal 1, which is often initiated by engagement of a member of the Toll-like receptor (TLR) family and which leads to increased expression of the IL-1ß mRNA and precursor protein, and signal 2, which triggers inflammasome assembly and cleavage of pro-IL-1ß [1,2]. IL-1ß plays important roles in several aspects of innate and adaptive immune responses and can contribute to the pathology of chronic inflammatory diseases when its production is dysregulated [3,4]. Of the NLRs, NLR-pyrin domain-containing 3 (NLRP3) is one of the best studied, and has been shown to induce inflammasome formation in response to a number of stimuli, including potassium efflux, lysosomal destabilization and generation of reactive oxygen species [1,5].

Inflammasome activation in response to pathogenic bacteria has been well studied and has been shown to involve the NLR-dependent sensing of microbial molecules such as flagellins, exotoxins and type III secretion system proteins [1,2]. Commensal bacteria, which often do not express such molecules, can also activate the inflammasome under some circumstances. For instance, IL-1ß is produced by mucosal mononuclear cells of patients with inflammatory bowel disease (IBD), a disorder in which intestinal inflammation is driven by gut commensals that are translocated across the damaged intestinal epithelium [6–8]. Despite the importance of commensal-induced IL-1ß in such situations, not much is known about the mechanisms of inflammasome activation that are involved. A recent study addressed this issue using a mouse model of IBD. The results indicated that the induction of colitis was associated with the commensal-dependent production of IL-1ß by inflammatory monocytes recruited to the intestinal lamina propria [9]. By screening a number of commensal bacteria, the investigators identified a strain of the pathobiont *Proteus mirabilis* as the only one able to elicit a robust IL-1ß response from mouse macrophages, and showed that a hemolysin expressed by this organism was involved in activating the NLRP3 inflammasome [9]. While these findings provide important new insights into the mechanisms of inflammasome activation, it is not clear whether they apply widely to more typical intestinal commensal bacteria, most of which are unlikely to produce hemolysins or similar cytotoxins.

We recently carried out experiments in which a human macrophage cell line was infected with commensal bacteria, the Gram-positive *Bifidobacterium longum* subspecies *infantis* (which we will refer to as *B*. *infantis*) and the Gram-negative *Bacteroides fragilis*, representing some of the most abundant organisms present in the intestinal microbiota of the human infant and adult, respectively [10]. Neither *B*. *infantis* nor *B*. *fragilis* produces the typical molecules that have been implicated in inflammasome activation, including flagellins, exotoxins or type III secretion system proteins. Furthermore, only *B*. *fragilis* expresses lipopolysaccharide (LPS), which could, in theory, activate the so-called non-canonical inflammasome, a recently discovered complex that senses and responds to cytosolic LPS [1,11]. Nevertheless, we found that both *B*. *infantis* and *B*. *fragilis* induced secretion of IL-1ß by mouse and human macrophages, indicating a role for inflammasome activation in the response to common commensals. Since there is nothing known about how these bacteria induce IL-1ß secretion, we carried out the experiments reported here to shed light on the mechanisms involved.

## Materials and Methods

### Infection of mouse macrophages with *B*. *infantis* and *B*. *fragilis*

Murine bone marrow-derived macrophages (BMDMs) were prepared as previously described [12]. In brief, bone marrow from wild-type (WT) C57BL/6 mice (Jackson Laboratory, Bar Harbor, ME) was cultured for 6–7 days in 24-well tissue culture plates in RPMI 1640 medium containing 10% heat-inactivated fetal bovine serum and antibiotics and supplemented with 10% L929 conditioned medium. The cells were washed and placed in antibiotic- and serum-free medium before carrying out infections. *B*. *infantis* (strain S12) and *B*. *fragilis* (strain NCTC 9343) were obtained from the American Type Culture Collection, Manassas, VA and were provided by Dr. Deepak Vijaykumar, Massachusetts General Hospital. The bacteria were grown anaerobically at 37°C for 20–22 hours in broth cultures of dMan Rogosa Sharpe (MRS) medium and reinforced Clostridial medium (RCM), respectively. Both media were prepared according to recommendations provided by the manufacturer (Becton-Dickinson, Franklin Lakes, NJ). In some experiments, the bacteria were physically separated from the macrophages by using Transwell inserts with a 0.4 micron membrane (Corning Corp., Corning, NY), with the bacteria added to the upper chamber of the insert. Infection of the macrophages with *Citrobacter rodentium* (strain DBS100, kindly provided by Dr. Hai Ning Shi, Massachusetts General Hospital) was used as a positive control for IL-1ß production in some of the studies. The bacteria were grown and prepared for infection as described earlier [13]. Heat killing of the *B*. *infantis* and *B*. *fragilis* was carried out by placing suspensions of the bacteria in a 95°C water bath for 30 minutes. Plating of an aliquot of the bacterial suspensions after the heat treatment indicated > 99% reduction in viability. Fifty million colony forming units (cfu) of *B*. *infantis* and *B*. *fragilis* (or equivalent numbers of the heat-killed bacteria) were suspended in antibiotic- and serum-free RPMI 1640 and added to the BMDM (multiplicity of infection 50:1). The infection was allowed to proceed for 1 hour at 37°C. After washing the cells and refreshing the medium, incubation was continued overnight before collecting the supernatants for analysis. In some experiments, the cells were incubated for 4 hours after the infection, then washed and lysed in Trizol reagent (Life Technologies, Grand Island, NY) for RNA preparation. Immortalized mouse BMDM lines generated from WT and NLRP3 knockout (KO) mice were kindly provided by Dr. Katherine Fitzgerald, University of Massachusetts Medical School [14,15]. Similar immortalized macrophages from a variety of KO mice have been used widely in the field to investigate the requirement for different genes in the innate immune response, and behave similarly to primary macrophages [14–16]. The WT and NLRP3 KO immortalized macrophage lines were maintained in Dulbecco’s modified Eagle medium (DMEM) with 10% heat-inactivated fetal bovine serum and antibiotics, and were infected with *B*. *infantis* and *B*. *fragilis* exactly like the primary BMDMs.

### Infection of THP-1 macrophages

Experiments with THP-1 cells were carried out as described in detail earlier [17]. In brief, the human monocyte cell line THP-1 (American Type Culture Collection, Manassas, VA) was maintained at 37°C in 5% CO_2_ in RPMI 1640 medium supplemented with 10% heat-inactivated fetal bovine serum and antibiotics. The cells were differentiated into macrophages by treatment with 50 ng/ml of phorbol myristate acetate for 72 hours. The differentiated cells were placed in serum- and antibiotic-free medium and then infected with *B*. *infantis* and *B*. *fragilis* as described above. After 1 hour at 37°C and 5% CO_2_, the cells were washed and incubated for a further 4 hours in fresh medium before collecting the supernatants for analysis.

### Preparation of primary human monocyte-derived macrophages (MDM)

Blood anticoagulated with acid citrate dextrose was obtained aseptically from healthy human volunteers. Monocytes were isolated from the blood by density centrifugation on Ficoll-Hypaque (GE Healthcare, Pittsburgh, PA) followed by positive magnetic selection with anti-CD14 microbeads (Miltenyi Biotec, Auburn, CA) using previously published protocols [18,19]. The cells were cultured in RPMI 1640 medium with 10% heat-inactivated fetal bovine serum and antibiotics in the presence of 10 ng/ml of macrophage-colony stimulating factor (Peprotech, Rocky Hill, NJ) for 7 days to induce macrophage differentiation. The macrophages were placed in antibiotic- and serum-free medium prior to infections with *B*. *infantis* and *B*. *fragilis* for 1 hour as described above. The cells were washed and supernatants collected for analysis after a further 4 hours.

### Use of pharmacologic inhibitors

Cytochalasin D (Sigma-Aldrich, St. Louis, MO) was added to the cells 30 minutes before infection, remained in the medium during the 1 hour infection period and for different times after the infection as indicated in individual experiments. The concentration of cytochalasin D used was based on the results of a gentamicin protection assay in which macrophages were infected with *B*. *fragilis*, then washed and incubated for 1 hour in medium containing 200 μg/ml of gentamicin. After washing the cells again, cell lysates were prepared and serial dilutions plated on RCM agar to determine the number of surviving bacteria. Preliminary experiments showed that treatment with 200 μg/ml of gentamicin for 1 hour killed > 99% of the *B*. *fragilis*. Other inhibitors–potassium chloride (Sigma-Aldrich, St. Louis, MO), Z-YVAD-FMK (referred to hereafter as ZYVAD, EMD Millipore, Billerica, MA), ruthenium red (Tocris Bioscience, Minneapolis, MN), and apyrase (Sigma-Aldrich, St. Louis, MO)–were used at concentrations that were based on information in the literature. They were added to the cells 30 minutes before infection and were maintained in the medium for the times indicated in individual experiments.

### IL-1ß ELISA

IL-1ß concentrations in cell supernatants were measured with kits specific for the mouse or human cytokine using protocols provided by the manufacturer (eBioscience, San Diego, CA). The ELISA detected both the precursor and mature forms of IL-1ß. However, based on western blotting, cell supernatants contained the mature form predominantly, with little or no detectable precursor.

### Western blotting

Immunoblotting was carried out as previously described [17]. Cellular extracts or supernatants (equalized for total protein concentration) were separated by SDS-PAGE and subjected to semi-dry blotting onto a nitrocellulose membrane. The membrane was blocked and incubated overnight with an anti-caspase 1 antibody (Abcam, Cambridge, MA) or with an anti-IL-1ß antibody (R&D Systems, Minneapolis, MN) at 4°C. The blot was developed with a fluorescently-tagged secondary antibody and the signals were visualized with an Odyssey infra-red fluorescence imaging system (Li-Cor Biosciences, Lincoln, NE).

### Quantitative RT-PCR

Total RNA was prepared from Trizol extracts according to manufacturer’s recommendations. It was reverse transcribed with random hexamers and amplified with primers specific for IL-1ß and GAPDH or actin using methods similar to those described earlier [20–22]. Expression of the IL-1β transcript is shown relative to that of GAPDH or actin using the 2^-ΔCt^ method. The sequences of the IL-1ß, GAPDH and actin primers have been published previously [17,20,21].

### Cytotoxicity assay

Cell viability was assessed using the CytoTox 96 kit (Promega, Madison, WI), which measures the proportion of cellular lactate dehydrogenase (LDH) released into the supernatant.

### Statistical analysis

The results are displayed as the means +/- standard deviation with each figure representing the results of at least 3, and in most cases 6, biological replicates, i.e., at least 3 independent infections or stimulations. The raw data used to calculate the means and standard deviations for each figure are provided in tabular form in the supplementary information file (S1 File). The two-tailed, unpaired student’s *t* test was used to assess significance, with a *p* value < 0.05 being considered statistically significant. Statistically significant differences are indicated with asterisks in the figures, while the corresponding *p* and n values are specified in the figure legends.

### Ethics statement

Animal studies were carried out in strict accordance with the recommendations in the Guide for the Care and Use of Laboratory Animals of the National Institutes of Health. Bone marrow was harvested from mice after euthanasia by controlled-flow carbon dioxide asphyxia as per recommended guidelines. All efforts were made to minimize animal suffering. The experiments were reviewed and approved by the Institutional Animal Care and Use Committee of Massachusetts General Hospital (protocol number 2008N000061, animal welfare assurance number A3596-01). Blood was collected from healthy human volunteers after obtaining written consent. Participant consent was documented by signature on a consent form. The human studies were reviewed and approved by the Institutional Review Board of Massachusetts General Hospital (protocol number 1999P007782, human subjects assurance number 00003136).

## Results

**Fig 1:**
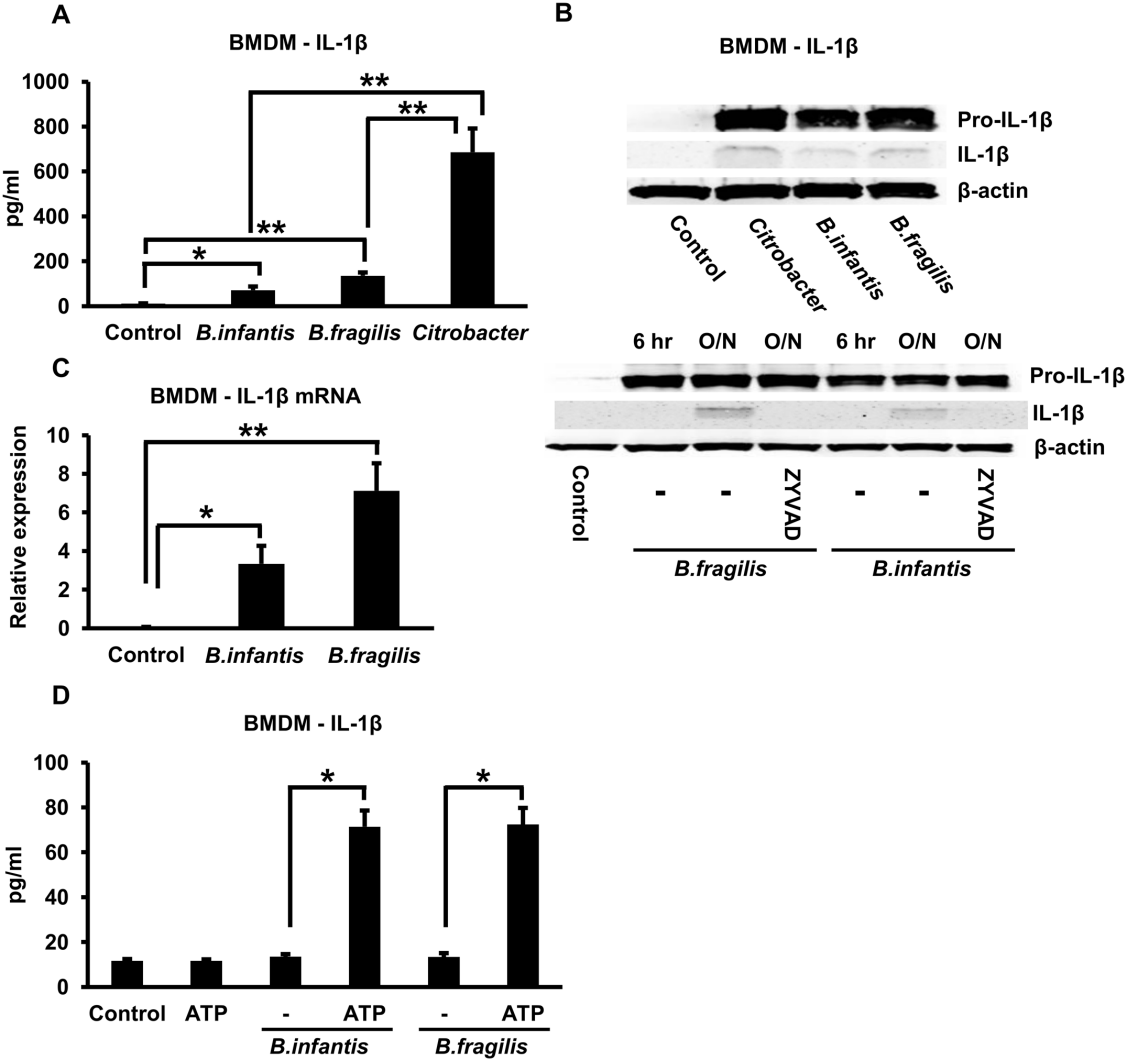
*B*. *infantis* and *B*. *fragilis* induce IL-1ß secretion in mouse BMDMs after overnight incubation. **A.** Mouse BMDMs were infected with *B*. *infantis*, *B*. *fragilis* or *C*. *rodentium* for 1 hour. The cells were washed and incubated overnight in fresh medium. IL-1ß concentrations in the supernatants were determined by ELISA. *p = 0.0003, **p < 0.0001, n = 6 per experimental condition. **B.** Mouse BMDMs were infected with *B*. *infantis*, *B*. *fragilis* or *C*. *rodentium* as in **A** and then incubated overnight (upper panel) or for the times indicated (6 hours or overnight–O/N–lower panel). Cell lysates and supernatants were immunoblotted to detect pro-IL-1ß and mature IL-1ß, respectively, while cell lysates were blotted with an anti-actin antibody to confirm equal loading of lanes. The caspase inhibitor ZYVAD was added to the cells at a concentration of 100 μM where indicated. **C.** Mouse BMDMs were infected with *B*. *infantis* or *B*. *fragilis* for 1 hour. The cells were washed, placed in fresh medium and incubated for a further 4 hours. Total cellular RNA was prepared and used to determine relative IL-1ß mRNA expression by qRT-PCR. *p = 0.0003, **p < 0.0001, n = 6 per experimental condition. **D.** Mouse BMDMs were infected with *B*. *infantis* for 1 hour, washed, then incubated in fresh medium for 4 hours in the presence or absence of 5 mM ATP added exogenously during the last 1 hour. Supernatants were collected at the end of the 4 hour incubation and were analyzed by ELISA to determine IL-1ß concentrations. *p < 0.0001, n = 6 per experimental condition.

Infection of mouse BMDMs with *B*. *infantis* or *B*. *fragilis* for 1 hour followed by overnight incubation resulted in the appearance of appreciable levels of IL-1ß in the cell supernatants as determined by ELISA (Fig 1A). The amounts of secreted IL-1ß induced by *B*. *infantis* and *B*. *fragilis* were significantly less than the amount induced by infection with *Citrobacter rodentium*, a Gram-negative bacterial enteropathogen that is known to activate the NLRP3 inflammasome (Fig 1A) [23]. The secretion of IL-1ß was not associated with alterations in cell viability as indicated by an LDH release assay (S1A Fig). Since the ELISA measured both the precursor and mature, processed forms of IL-1ß, we also carried out western blots to detect these forms in cell lysates and cell supernatants respectively. As shown in the upper panel of Fig 1B, infection with *B*. *infantis*, *B*. *fragilis* or *C*. *rodentium* induced an appreciable increase of the precursor pro-IL-1ß in the cell lysate, as well as an increase of the mature IL-1ß in the supernatant, after overnight incubation. The increase in pro-IL-1ß in the cell lysate was observed by 6 hours after infection with *B*. *infantis* or *B*. *fragilis* whereas the increase in the mature form in the supernatant was seen only after overnight incubation (Fig 1B, lower panel). Moreover, the *B*. *infantis*- and *B*. *fragilis*-induced appearance of mature IL-1ß in the cell supernatant was prevented by treating the cells with ZYVAD, a cell-permeable inhibitor of caspases 1, 4 and 5 (Fig 1B, lower panel), results that are consistent with the involvement of the inflammasome. The full-length blots corresponding to the cropped versions shown in Fig 1B are shown in S1B Fig and indicate that the majority of the IL-1ß in the cell supernatant consists of the mature form. Interestingly, secreted IL-1ß could not be detected in the cell supernatant 4 hours after the infection period even though there was a significant increase in the mRNA in the cells at this time (Fig 1C). However, if ATP, a known activator of the inflammasome [24,25], was exogenously added to the cells 3 hours after the infection period, secreted IL-1ß could be easily detected in the supernatants at 4 hours after the infection period (Fig 1D). Thus, these results indicate that in mouse macrophages, *B*. *infantis* and *B*. *fragilis* activated the signal leading to increased expression of the IL-1ß transcript (and the pro-IL-1ß precursor protein) fairly rapidly, whereas it delivered the signal required for inflammasome activation and IL-1ß secretion more slowly. *B*. *infantis* and *B*. *fragilis* infection also induced significant up-regulation of the NLRP3 mRNA after overnight incubation (S1C Fig).

**Fig 2:**
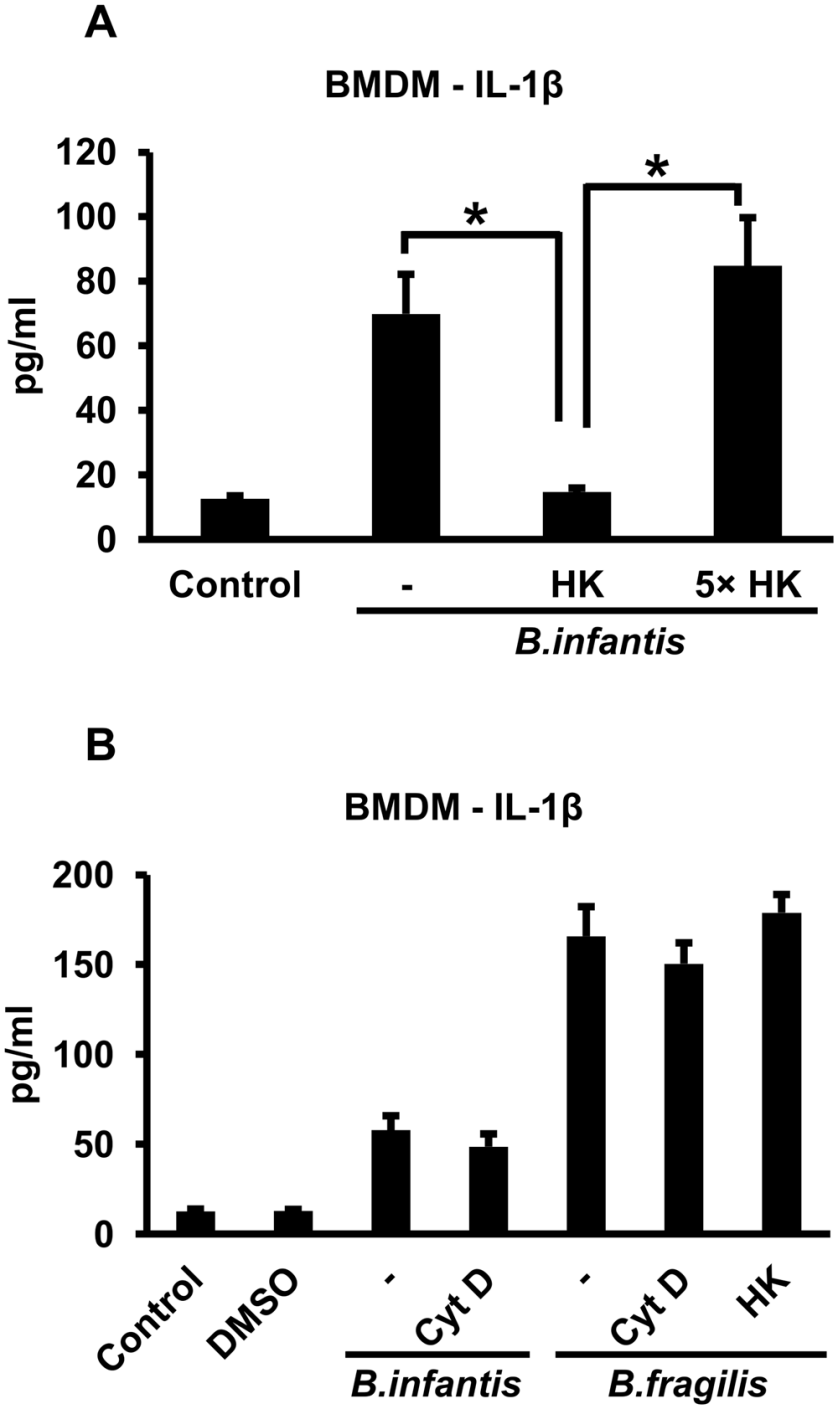
Bacterial viability and phagocytosis are not required for *B*. *infantis*- and *B*. *fragilis*-induced IL-1ß secretion by mouse BMDMs. **A.** Mouse BMDMs were infected with live *B*. *infantis* or heat-killed (HK) bacteria, either equivalent in number to the live organisms or a 5-fold excess (5X), for 1 hour. The cells were washed and incubated overnight in fresh medium. IL-1ß concentrations in the supernatants were determined by ELISA. In the condition with 5X heat-killed bacteria, the cells were exposed continuously to the bacteria overnight without the intervening wash. *p < 0.0001, n = 6 per experimental condition. **B.** Mouse BMDMs were infected with live *B*. *infantis* or *B*. *fragilis* or an equivalent number of heat-killed (HK) *B*. *fragilis* for 1 hour in the presence or absence of 5 μM cytochalasin D (CytD, dissolved in DMSO) as indicated. The cells were washed and incubated overnight in fresh medium, with cytochalasin D added back as appropriate. IL-1ß concentrations in the supernatants were determined by ELISA. n = 6 per experimental condition.

We then carried out experiments to elucidate how *B*. *infantis* and *B*. *fragilis* activate the inflammasome in mouse BMDMs. In contrast to the results with live bacteria, exposing the cells to an equivalent number of heat-killed *B*. *infantis* for 1 hour followed by overnight incubation did not result in IL-1ß secretion (Fig 2A). However, if the BMDMs were continuously exposed overnight to an approximately 5-fold higher number of heat-killed bacteria, IL-1ß secretion was restored to the level induced by live organisms (Fig 2A). Thus, the bacteria did not have to be viable to induce IL-1ß secretion as long as there was a sufficient number of them. We then went on to determine whether the bacteria had to be phagocytosed in order for inflammasome activation to occur. We used 5 μM cytochalasin D to inhibit phagocytosis, which resulted in > 95% reduction in the number of intracellular *B*. *fragilis* as determined by gentamicin protection assay (S2A Fig). We could not carry out a similar experiment with *B*. *infantis* since this organism is not killed by gentamicin, but we presume that cytochalasin is equally effective in inhibiting uptake of this organism. BMDMs were treated with cytochalasin D for 30 minutes and the cells were infected with *B*. *infantis* for 1 hour in the continued presence of cytochalasin D. After washing the cells, cytochalasin D was added back for the overnight duration of the experiment. Overnight supernatants were collected and IL-1ß concentrations determined. As shown in Fig 2B, the cytochalasin D did not significantly alter IL-1ß secretion in response to the *B*. *infantis* infection although a slight reduction was noted in most experiments. The cytochalasin D treatment did not affect cell viability as indicated by LDH release. We carried out similar experiments with *B*. *fragilis* and found that IL-1ß secretion by mouse BMDMs in response to this organism also did not require bacterial viability and was not significantly inhibited by cytochalasin D (Fig 2B). Interestingly, *B*. *fragilis* induced more robust IL-1ß secretion than *B*. *infantis*, and even equivalent numbers of the heat-killed bacteria elicited as much of the secreted cytokine as the live organisms (Fig 2B). Cytochalasin D did not inhibit IL-1ß secretion induced by heat-killed *B*. *infantis* or *B*. *fragilis* (S3A Fig).

**Fig 3:**
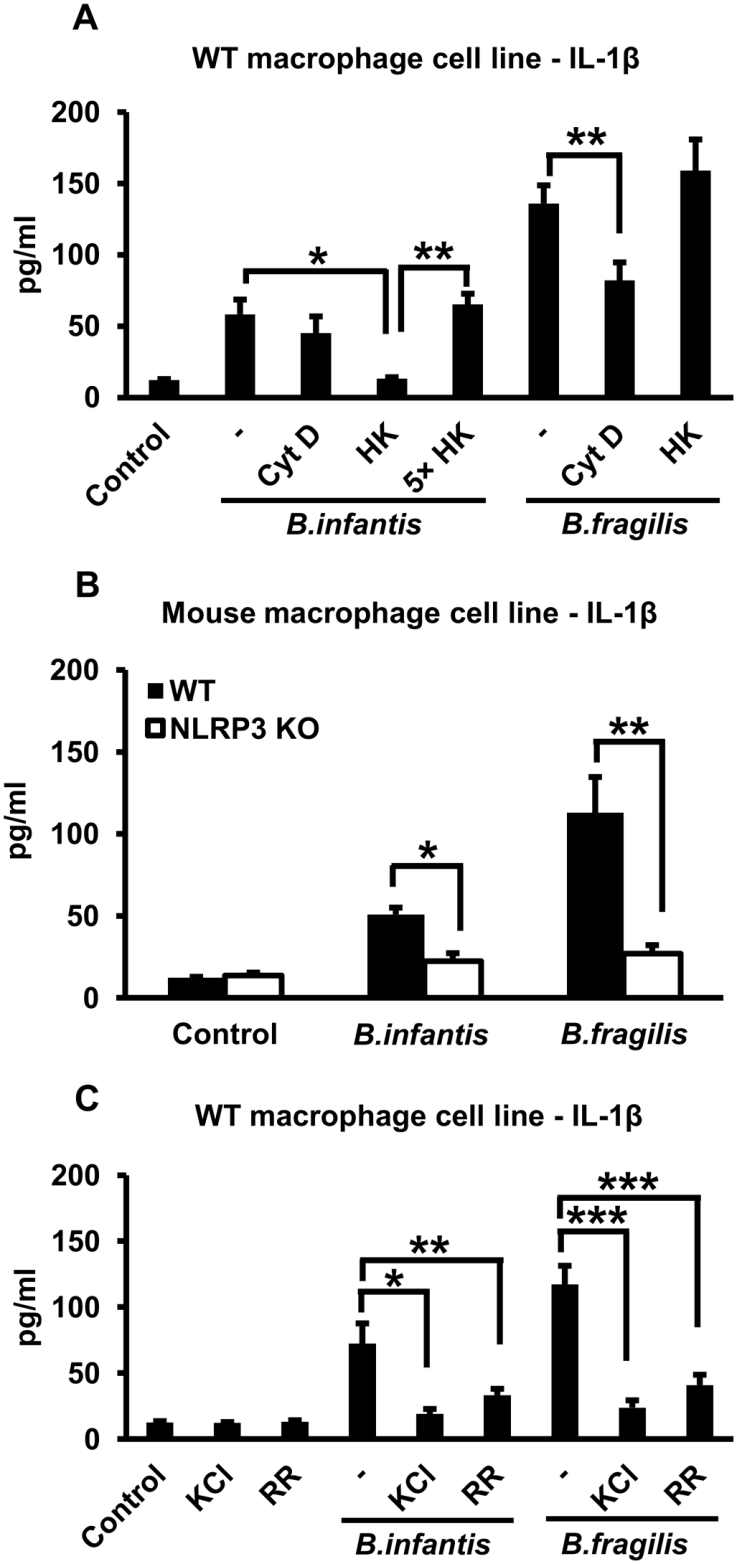
*B*. *infantis* and *B*. *fragilis* induce IL-1ß secretion in immortalized mouse macrophages in an NLRP3- and potassium efflux-dependent fashion. **A.** Immortalized WT mouse macrophages were infected with equivalent numbers of live or heat-killed (HK) *B*. *infantis* or *B*. *fragilis* (as well as a 5-fold higher number (5X) in the case of heat-killed *B*. *infantis*) for 1 hour in the presence or absence of 5 μM cytochalasin D (CytD) as indicated. The cells were washed and incubated overnight in fresh medium, with cytochalasin D added back as appropriate. IL-1ß concentrations in the supernatants were determined by ELISA. In the condition with 5X heat-killed bacteria, the cells were exposed continuously to the bacteria overnight without the intervening wash step. *p = 0.0001, **p < 0.0001, n = 6 per experimental condition. **B.** WT or NLRP3 KO immortalized mouse macrophages were infected with live *B*. *infantis* or *B*. *fragilis* for 1 hour. The cells were washed and incubated overnight in fresh medium. IL-1ß concentrations in the supernatants were determined by ELISA. *p < 0.0001, **p = 0.0001, n = 6 per experimental condition. **C.** Immortalized WT mouse macrophages were infected with live *B*. *infantis* or *B*. *fragilis* for 1 hour in the presence or absence of 50 mM potassium chloride (KCl) or 2 μM ruthenium red (RR) as indicated. The cells were washed and incubated overnight in fresh medium, with potassium chloride or ruthenium red added back as appropriate. IL-1ß concentrations in the supernatants were determined by ELISA. *p = 0.0002, **p = 0.0011, ***p < 0.0001, n = 6 per experimental condition.

We carried out similar experiments with an immortalized BMDM line derived from WT mice. As shown in Fig 3A, we found that infection with *B*. *infantis* or *B*. *fragilis* for 1 hour induced IL-1ß secretion after overnight incubation, and that bacterial viability was not required for this process (although, as for the primary BMDMs, continuous exposure to a 5-fold excess of heat-killed bacteria was needed in the case of *B*. *infantis*). Treating the cells with cytochalasin D had little or no effect on IL-1ß secretion induced by *B*. *infantis* and reduced the response to *B*. *fragilis* by about 50% (Fig 3A), whereas cytochalasin D inhibited the phagocytic uptake of *B*. *fragilis* by > 95% (S2B Fig). Unlike the WT cell line, an immortalized BMDM line derived from NLRP3 KO mice did not secrete IL-1ß in response to infection with live *B*. *infantis* or *B*. *fragilis* (Fig 3B), indicating the involvement of the NLRP3 inflammasome. We also found that if the WT macrophage line was exposed to elevated extracellular potassium concentrations (50 mM potassium chloride) or the channel blocker ruthenium red, IL-1ß secretion in response to *B*. *infantis* or *B*. *fragilis* was significantly reduced (Fig 3C). Raising extracellular potassium concentration also inhibited IL-1ß secretion by BMDMs in response to heat-killed *B*. *infantis* or *B*. *fragilis* (S3A Fig). Thus, activation of the NLRP3 inflammasome by these bacteria is dependent on potassium efflux.

**Fig 4:**
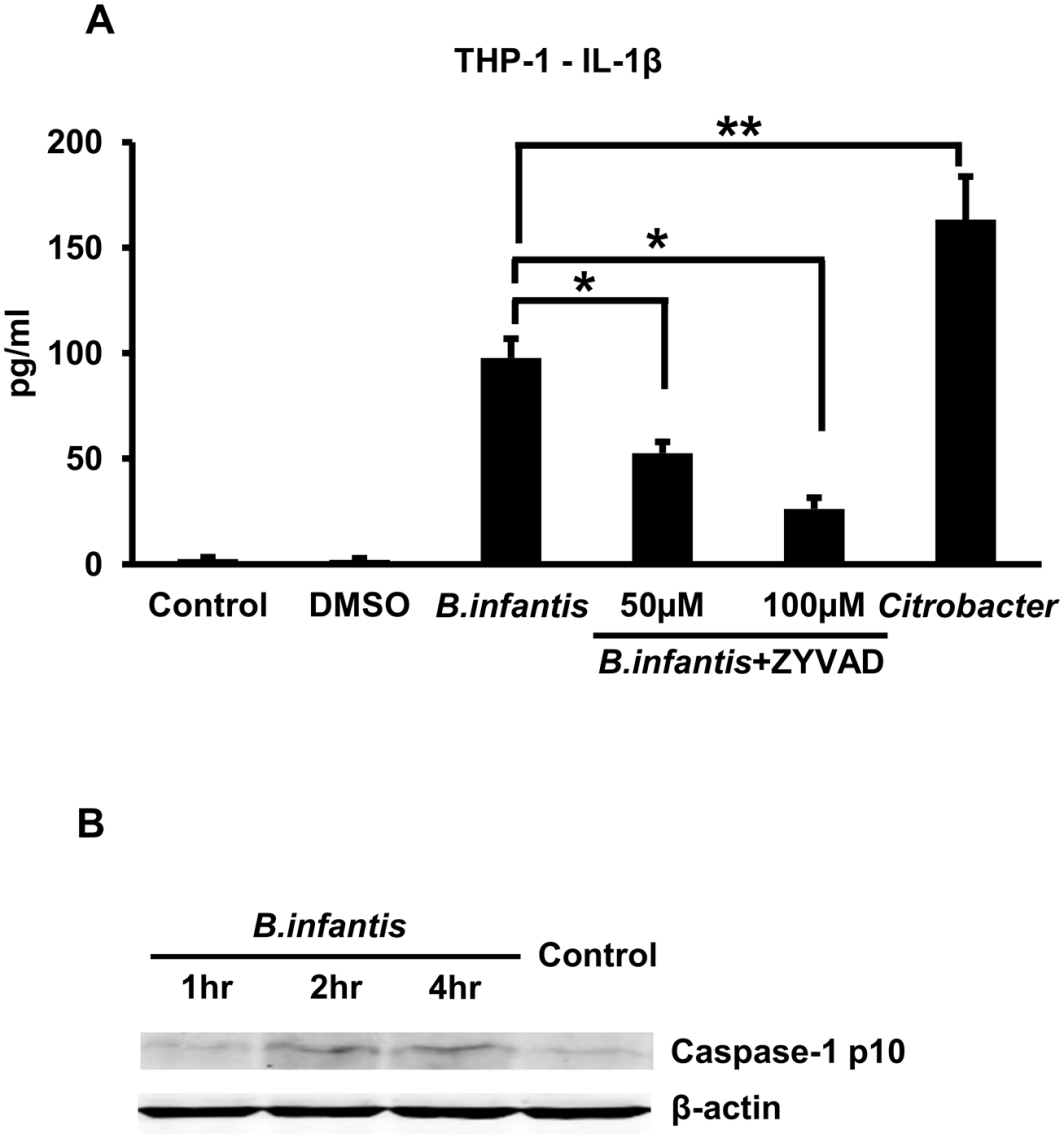
*B*. *infantis*-induced IL-1ß secretion by THP-1 macrophages involves caspase 1. **A.** THP-1 macrophages were infected with *B*. *infantis* or *C*. *rodentium* for 1 hour, washed, then incubated in fresh medium for 4 hours. ZYVAD at a final concentration of 50 or 100 μM was present in the medium, as indicated, throughout the course of the experiment. Supernatants were collected at the end of the 4 hour incubation and IL-1ß concentrations determined by ELISA. *p < 0.0001, **p = 0.0002, n = 6 per experimental condition. **B.** THP-1 macrophages were infected with *B*. *infantis* for 1 hour, washed, then incubated in fresh medium for the times indicated before preparation of cell lysates. The lysates were analyzed by western blotting with an antibody to caspase 1, with equal loading of lanes being confirmed by blotting with an anti-ß-actin antibody. The p10 processed form of caspase 1 is shown.

We proceeded to investigate how *B*. *infantis* and *B*. *fragilis* induced IL-1ß secretion in human macrophages. Infection of THP-1 macrophages with *B*. *infantis* for 1 hour resulted in robust secretion of IL-1ß over the course of 4 hours, although the amount was significantly less than that secreted in response to *Citrobacter* infection (Fig 4A). *B*. *infantis*-induced IL-1ß secretion was significantly reduced in a dose-dependent manner by treating the cells with ZYVAD, an inhibitor of caspases 1, 4 and 5 (Fig 4A). Consistent with the involvement of caspase 1 in IL-1ß secretion induced by this commensal, we found that the infection led to increased levels of the processed p10 form of the enzyme as detected by western blotting (Fig 4B, the corresponding uncropped blots are shown in S4 Fig). These observations are in keeping with the idea that *B*. *infantis* activates the inflammasome in human macrophages. Based on the results of an LDH release assay, neither infection with the bacteria nor the ZYVAD treatment was associated with significant changes in cell viability.

**Fig 5:**
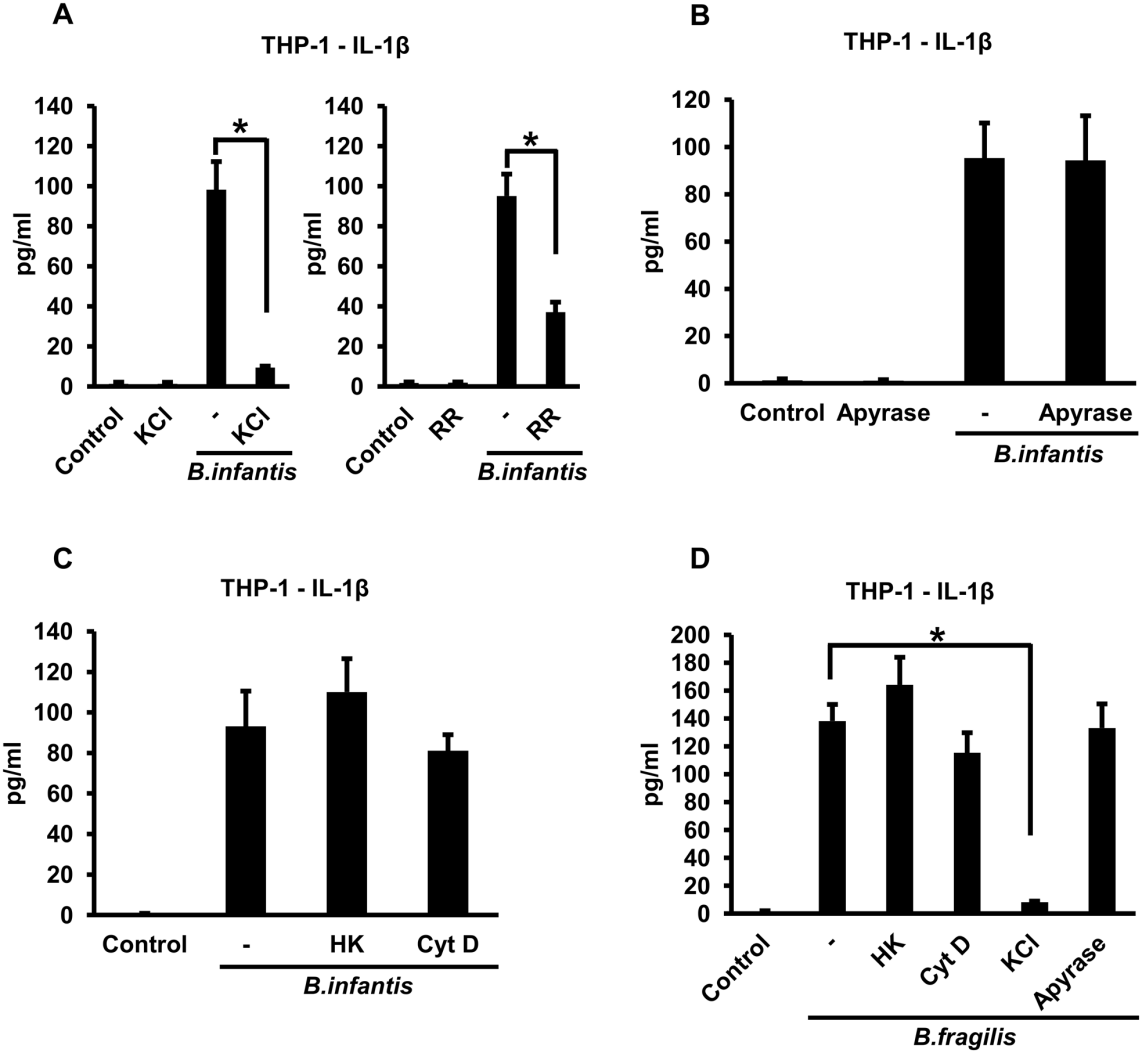
*B*. *infantis*- and *B*. *fragilis*-induced IL-1ß secretion by THP-1 macrophages is dependent on potassium efflux but does not require bacterial viability or phagocytosis. **A.** THP-1 macrophages were infected with *B*. *infantis* for 1 hour in the presence or absence of 50 mM potassium chloride (KCl) or 2 μM ruthenium red (RR), as indicated. The cells were washed and incubated in fresh medium, with potassium chloride or ruthenium red added back as appropriate. Supernatants were collected at the end of 4 hours and IL-1ß concentrations determined by ELISA. *p < 0.0001, n = 6 per experimental condition. **B.** THP-1 macrophages were infected with *B*. *infantis* for 1 hour. The cells were washed and incubated in fresh medium, with 5 units/ml of apyrase added as indicated. Supernatants were collected after 4 hours and IL-1ß concentrations determined by ELISA, n = 6 per experimental condition. **C.** THP-1 macrophages were infected with equivalent numbers of live or heat-killed (HK) *B*. *infantis* for 1 hour, in the presence of 5 μM cytochalasin D (CytD) where indicated. The cells were washed and incubated in fresh medium, with cytochalasin D added back as appropriate. Supernatants were collected after 4 hours and IL-1ß concentrations determined by ELISA, n = 6 per experimental condition. **D.** THP-1 macrophages were infected with equivalent numbers of live or heat-killed (HK) *B*. *fragilis* for 1 hour, in the presence of 5 μM cytochalasin D (CytD) or 50 mM potassium chloride (KCl) where indicated. The cells were washed and incubated in fresh medium, with cytochalasin D, potassium chloride or 5 units/ml of apyrase added back as appropriate. Supernatants were collected after 4 hours and IL-1ß concentrations determined by ELISA. *p < 0.0001, n = 6 per experimental condition.

To determine if the NLRP3 inflammasome might be involved in *B*. *infantis*-induced IL-1ß secretion by THP-1 macrophages, we inhibited potassium efflux, one of the mechanisms by which this inflammasome is activated [1], by adding potassium chloride to the extracellular medium at a final concentration of 50 mM. The elevated extracellular potassium markedly inhibited IL-1ß secretion (Fig 5A, left panel). A similar, albeit less dramatic, reduction in *B*. *infantis*-induced IL-1ß secretion was seen when the cells were treated with the channel blocker ruthenium red (Fig 5A, right panel). These results are consistent with involvement of the NLRP3 inflammasome in IL-1ß secretion by THP-1 macrophages in response to *B*. *infantis* infection. One mechanism by which potassium efflux might be induced during the course of infection with *B*. *infantis* is via activation of the P2X7 purinergic receptor by extracellular ATP [21,22]. To test this idea, we examined the effect of the ATP degrading enzyme apyrase. Addition of apyrase to the medium did not inhibit *B*. *infantis*-induced IL-1ß secretion by the THP-1 macrophages (Fig 5B), suggesting that release of endogenous ATP is not involved in this process. Note that this result does not contradict the findings in Fig 1D since apyrase was used in the experiment of Fig 5B to determine the role of endogenously released ATP in IL-1ß secretion, whereas exogenous ATP was added in the experiment of Fig 1D as a positive control for inflammasome activation. We then went on to elucidate the initial steps involved in the *B*. *infantis*-induced activation of the inflammasome in human macrophages. Heat killing the bacteria did not significantly affect their ability to induce IL-1ß secretion by the THP-1 cells (Fig 5C), indicating that the organisms did not need to be viable. Interestingly, unlike in the experiments with mouse macrophages, the number of heat-killed *B*. *infantis* did not need to be increased in order to elicit IL-1ß secretion equivalent to live bacteria. We also found that treating the cells with cytochalasin D had little or no effect on IL-1ß secretion induced by *B*. *infantis* (Fig 5C), indicating that phagocytosis was not required. We carried out similar experiments with *B*. *fragilis* and found that IL-1ß secretion by THP-1 macrophages in response to this organism was also markedly inhibited by increasing extracellular potassium but was not significantly affected by heat killing the bacteria, by cytochalasin D or by apyrase (Fig 5D). Cytochalasin D did not inhibit IL-1ß secretion induced by heat-killed *B*. *infantis* or *B*. *fragilis*, whereas raising extracellular potassium concentration did (S3B Fig).

**Fig 6:**
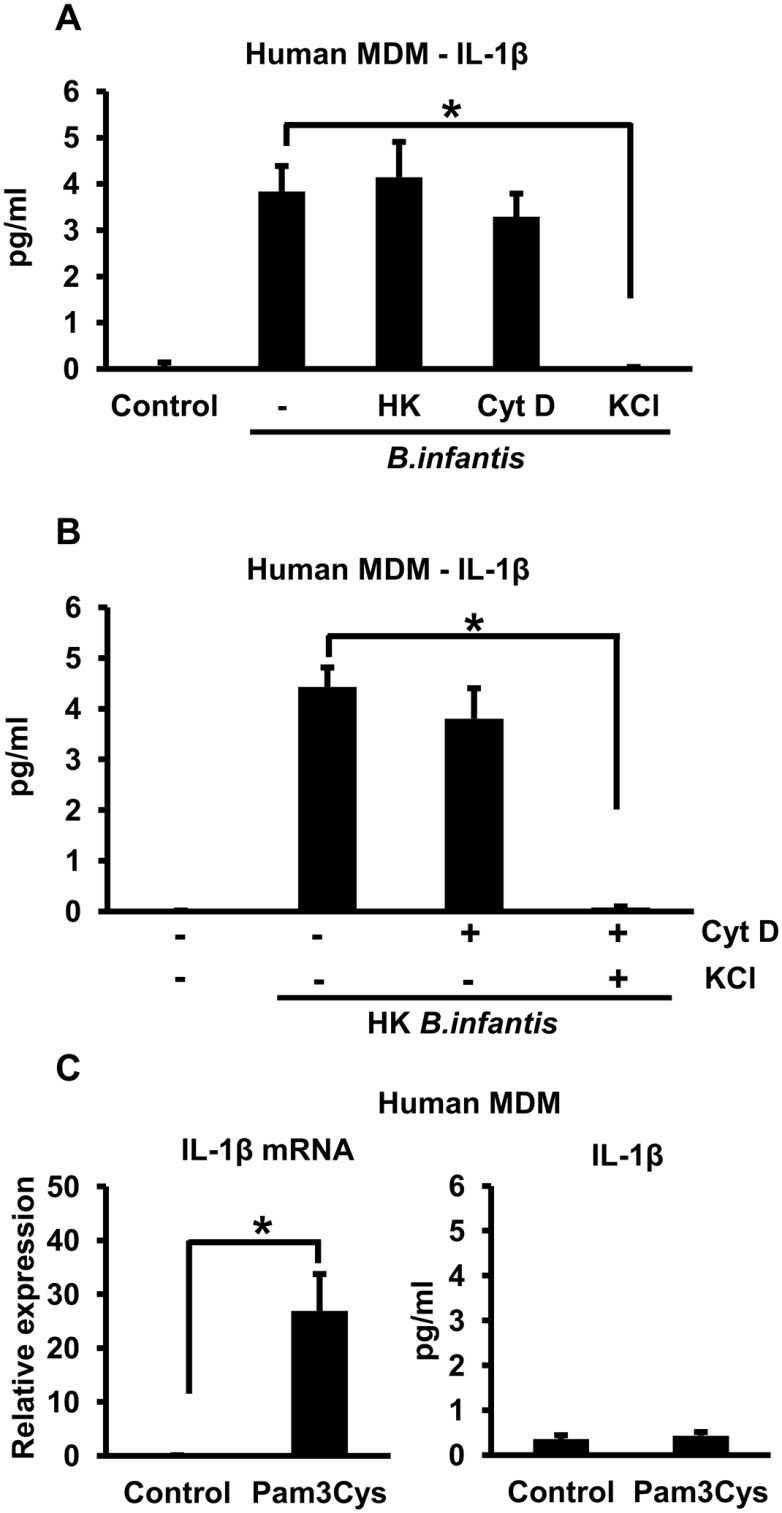
*B*. *infantis*-induced IL-1ß secretion by primary human MDMs. **A.** Primary human MDMs were infected with equivalent numbers of live or heat-killed (HK) *B*. *infantis* for 1 hour in the presence or absence 5 μM cytochalasin D (Cyt D) or 50 mM potassium chloride (KCl) as indicated. The cells were washed, then incubated in fresh medium, with potassium chloride added back as appropriate. Supernatants were collected after 4 hours and IL-1ß concentrations determined by ELISA. *p < 0.0001, n = 6 per experimental condition. **B.** Primary human MDMs were infected with heat-killed *B*. *infantis* for 1 hour in the presence or absence 5 μM cytochalasin D (Cyt D) or 50 mM potassium chloride (KCl) as indicated. The cells were washed, then incubated in fresh medium, with potassium chloride added back as appropriate. Supernatants were collected after 4 hours and IL-1ß concentrations determined by ELISA. *p < 0.0001, n = 6 per experimental condition. **C.** Primary human MDMs were treated with 10 μg/ml of Pam3Cys for 4 hours, after which cellular RNA was analyzed by quantitative RT-PCR to determine IL-1ß mRNA levels (left panel) and supernatants were analyzed by ELISA to determine secreted IL-1ß concentrations (right panel). *p = 0.0002, n = 6 per experimental condition.

To substantiate our findings with THP-1 cells, we repeated some of the key experiments using primary human peripheral blood MDMs. Although IL-1ß secretion by these cells was considerably less than by THP-1 macrophages, it was clearly induced by infection with *B*. *infantis*. As in the THP-1 cells, *B*. *infantis*-induced IL-1ß secretion by primary human MDMs was not affected by heat killing the bacteria or by inhibiting phagocytosis with cytochalasin D, but was almost completely inhibited by raising the levels of extracellular potassium (Fig 6A). These results suggested that surface interactions between the bacteria and the macrophage plasma membrane were sufficient to induce potassium efflux and thus activate the inflammasome. To substantiate this idea, we showed that exposing cytochalasin D-treated MDMs to elevated extracellular potassium eliminated IL-1ß secretion induced by heat-killed *B*. *infantis* (Fig 6B). To determine whether inflammasome activation under these circumstances might simply reflect triggering of a TLR, we treated the cells with Pam3Cys, a ligand for TLR2, the TLR most likely to be involved in responding to the Gram-positive, non-flagellated *B*. *infantis*. Although stimulation of the MDMs with Pam3Cys resulted in significant up-regulation of the IL-1ß mRNA, it did not lead to appreciable IL-1ß secretion (Fig 6C). Thus, TLR2 is unlikely to be involved in *B*. *infantis*-induced triggering of the second signal required for inflammasome activation.

## Discussion

The gastrointestinal tract is home to a large and diverse community of commensal microorganisms that constitutes the gut microbiota [10]. When the intestinal epithelial barrier is damaged, these otherwise harmless organisms can come into contact with innate and adaptive immune cells in the gut-associated lymphoid tissues and activate potentially damaging inflammatory responses, including the production of IL-1ß. Despite the importance of commensal-induced IL-1ß secretion in the pathogenesis of intestinal inflammation, including in conditions like IBD [6–8], little is known about the mechanisms involved. Much of what we understand about how bacteria activate the inflammasome, the key protein complex required for processing and secretion of IL-1ß, is based on experiments with pathogens [1,2]. In most of these pathogen-based studies, the inflammasome was shown to be activated by specific bacterial molecules that entered the cytosol and interacted with NLRs, or by bacterial toxins that had membrane pore forming or other activities [2,26]. The results of a recent investigation suggested that similar mechanisms could be involved in inflammasome activation by commensals. Of several bacteria examined in that study, only a rather uncommon commensal–the pathobiont *Proteus mirabilis*–was able to induce appreciable amounts of IL-1ß secretion by mouse BMDM over the course of 3 hours [9]. This ability was linked to the expression of a cytotoxin, the hemolysin HpmA, that promoted potassium efflux and thus activated the NLRP3 inflammasome [9]. Although more typical commensals–species of *Bacteroides* and *Clostridium*–were also studied, they were found not to induce detectable IL-1ß secretion, possibly because of the relatively short 3-hour period of incubation [9].

Our experiments with mouse macrophages (primary BMDMs and immortalized cell lines) indicate that activation of the NLRP3 inflammasome is involved in IL-1ß secretion induced by *B*. *infantis* and *B*. *fragilis*. The activation was dependent on potassium efflux and was resistant to heat killing of the bacteria (although a 5-fold higher number of killed bacteria was required in the case of *B*. *infantis*). Cytochalasin D treatment resulted in some reduction in IL-1ß secretion, which reached the level of statistical significance only in the case of the WT immortalized macrophage line, suggesting that the requirement for phagocytosis for *B*. *infantis*- and *B*. *fragilis*-induced inflammasome activation in mouse macrophages is at most partial. Similar characteristics were observed with regard to *B*. *infantis*- and *B*. *fragilis*-induced IL-1ß secretion by human macrophages. The requirement for potassium efflux in both THP-1 macrophages and primary human MDMs is consistent with involvement of the NLRP3 inflammasome [1]. Moreover, the induction of IL-1ß secretion by these cells was unaffected by heat killing of the bacteria and did not require phagocytosis. Taken together, our results from both mouse and human macrophages suggest that interactions between heat-resistant molecules on the bacterial surface and presumptive receptors on the macrophage plasma membrane are sufficient to induce potassium efflux and thus activate the inflammasome. Thus, the binding of *B*. *infantis* and *B*. *fragilis* to the surface of the macrophage appears to be the trigger that induces IL-1ß secretion. Indeed, we found that large numbers of both types of bacteria were bound to the surface of mouse and human macrophages (S2D and S2E Fig), about 100-fold greater than were phagocytosed (compare with S2A and S2B Fig, respectively). Furthermore, direct contact between the bacteria and the macrophages was required–if the two were separated by a 0.4 micron membrane in a Transwell system, induction of both IL-1ß mRNA and IL-1ß secretion were markedly inhibited (S5A, S5B, S5C and S5D Fig). This result also excludes a significant role for bacterial secreted molecules in inflammasome activation, which is consistent with the fact that heat-killed bacteria were able to induce IL-1ß secretion.

Our observations suggest that inflammasome activation results from the sensing of bacterial surface molecules by receptors on the macrophage plasma membrane. One potential mechanism for such sensing may involve the recognition of bacterial glycans by macrophage lectin receptors. Precedent for this idea is provided by studies showing that fungal β-glucans and mycobacterial glycolipids can signal through C-type lectin receptors to activate the NLRP3 inflammasome [27–29]. There is disagreement about the role of phagocytosis in lectin receptor-induced inflammasome activation, with some studies demonstrating a requirement for particle uptake while others support a phagocytosis-independent process [27,29,30]. Other mechanisms that might mediate inflammasome activation without the need for phagocytosis involve recognition of microbial ligands by the α5ß1 integrin and stimulation of mechanosensitive potassium channels as a result of plasma membrane perturbations caused by particle binding [31–33]. Scavenger receptors could also play a role since they have the ability to sense a variety of Gram-positive and Gram-negative organisms and to activate the NLRP3 inflammasome via induction of potassium efflux or the generation of reactive oxygen species [34–36]. Further studies will be required to clarify which of these possible mechanisms is responsible for inflammasome activation in macrophages in response to *B*. *infantis* and *B*. *fragilis*. It will also be relevant to find an explanation for the difference in kinetics of inflammasome activation by these bacteria in mouse and human macrophages.

The nature of the molecule on the surface of *B*. *infantis* and *B*. *fragilis* that is involved in activating the inflammasome remains a matter for speculation at this time. Based on our findings, we could imagine that the trigger for inflammasome activation is a surface molecule (protein, glycan or lipid) that is expressed at relatively low levels on *B*. *infantis* and at higher levels on *B*. *fragilis*. Thus, in order to activate the inflammasome, a higher number of killed *B*. *infantis* would be required compared to live organisms (which would be able to proliferate to the required numbers over the course of the experiment). Presumably, *B*. *fragilis* expresses sufficiently high levels of the relevant surface molecule so that even the starting number of live or killed bacteria is enough for inflammasome activation. Since *B*. *fragilis* is a Gram-negative organism, it is possible that it may differ from the Gram-positive *B*. *infantis* in having higher levels of surface glycolipids that are associated with the outer membrane. Thus, it is possible that the inflammasome-activating molecule may be a glycolipid.

Experiments in mouse models of IBD have yielded discordant results regarding the involvement of IL-1ß. Some studies have shown that neutralization or deficiency of IL-1ß reduces the severity of intestinal inflammation [9,37,38]. On the other hand, a recent study found that IL-1ß KO mice had more severe colitis than WT animals, indicating that the cytokine provides protection against intestinal inflammation, possibly by promoting epithelial repair [39]. There is similar controversy regarding the role of the inflammasome in mouse models of IBD, with some studies demonstrating that deficiency of NLRP3 or caspase 1 reduced the severity of colitis while others showed the opposite effect [40–43]. Some of these discrepancies have been suggested to be the result of variations in the gut microbiota [43]. In contrast to the work with mice, most human studies are consistent with the idea that IL-1ß plays a pro-inflammatory role in IBD and that it is an important contributor to the pathogenesis of this condition. Increased secretion of the cytokine has been observed in mucosal mononuclear cells from patients with Crohn’s disease and is also linked to genetic variants that heighten Crohn’s disease risk [7,44]. Significantly, a recent trial of IL-1 receptor blockade in a small number of patients with chronic granulomatous disease-associated colitis, a condition that has similarities to IBD, produced improvement in clinical and laboratory parameters [45]. Thus, further elucidation of the mechanisms by which commensal bacteria activate the inflammasome and induce IL-1ß secretion could lead to the development of new strategies to block this process and thus alleviate the intestinal inflammation associated with IBD. The observations reported here provide the foundation for such investigations.

## Supporting Information

S1 Fig Effects of *B*. *infantis* and *B*. *fragilis* on mouse BMDMs. **A.** Mouse BMDMs were infected with *B*. *infantis* and *B*. *fragilis* for 1 hour. The cells were washed and incubated overnight in fresh medium. Cytotoxicity was determined based on the proportion of cellular LDH released. n = 3 per experimental group. **B.** The full-length, uncropped blots corresponding to Fig 1B. **C.** Mouse BMDMs were infected with *B*. *infantis* and *B*. *fragilis* for 1 hour. The cells were washed and incubated overnight in fresh medium. Total RNA was prepared and NLRP3 expression was determined by qRT-PCR. *p = 0.0203, **p = 0.0276, n = 3 per experimental group.
(PDF)

S2 Fig Phagocytosis and binding of bacteria to macrophages. **A.** Mouse BMDMs were infected with *B*. *fragilis* for 1 hour in the presence of 5 μM of cytochalasin D (CytD) or an equivalent volume of DMSO, as indicated. The cells were washed and incubated for 1 hour in the presence of 200 μg/ml of gentamicin, with cytochalasin D or DMSO added back as appropriate. The cells were washed again, lysed and serial dilutions of the lysates plated to determine the numbers (colony forming units, CFU) of surviving *B*. *fragilis*. *p = 0.0088, n = 3 per experimental group. **B.** The WT immortalized macrophage cell line was infected with *B*. *fragilis* for 1 hour in the presence of 5 μM of cytochalasin D (CytD) or an equivalent volume of DMSO, as indicated. The cells were washed and incubated for 1 hour in the presence of 200 μg/ml of gentamicin, with cytochalasin D or DMSO added back as appropriate. The cells were washed again, lysed and serial dilutions of the lysates plated to determine the numbers (colony forming units, CFU) of surviving *B*. *fragilis*. *p = 0.0092, n = 3 per experimental group. **C.** THP-1 macrophages were infected with *B*. *fragilis* for 1 hour in the presence of 5 μM of cytochalasin D (CytD) or an equivalent volume of DMSO, as indicated. The cells were washed and incubated for 1 hour in the presence of 200 μg/ml of gentamicin, with cytochalasin D or DMSO added back as appropriate. The cells were washed again, lysed and serial dilutions of the lysates plated to determine the numbers (colony forming units, CFU) of surviving *B*. *fragilis*. *p = 0.0043, n = 3 per experimental group. **D.** Mouse BMDMs were infected with *B*. *infantis* or *B*. *fragilis* in the presence or absence of 5 μM of cytochalasin D (CytD) as indicated. The cells were washed, lysed and serial dilutions of the lysates plated to determine the numbers (colony forming units, CFU) of surviving *B*. *infantis* and *B*. *fragilis*. n = 3 per experimental group. **E.** THP-1 macrophages were infected with *B*. *infantis* or *B*. *fragilis* in the presence or absence of 5 μM of cytochalasin D (CytD) as indicated. The cells were washed, lysed and serial dilutions of the lysates plated to determine the numbers (colony forming units, CFU) of surviving *B*. *infantis* and *B*. *fragilis*. n = 3 per experimental group.
(PDF)

S3 Fig Involvement of phagocytosis and potassium efflux in IL-1ß secretion induced by heat-killed *B*. *infantis* and *B*. *fragilis*. **A.** Mouse BMDMs were exposed to heat-killed (HK) *B*. *infantis* or *B*. *fragilis* for 1 hour in the presence of 5 μM cytochalasin D (CytD) or 50 mM potassium chloride (KCl) as indicated. The cells were washed and incubated overnight in fresh medium with the cytochalasin D or potassium chloride added back as appropriate. IL-1ß concentrations in the supernatants were determined by ELISA. *p = 0.0002, **p = 0.0001, n = 3 per experimental group. **B.** THP-1 macrophages were exposed to heat-killed (HK) *B*. *infantis* or *B*. *fragilis* for 1 hour in the presence of 5 μM cytochalasin D (CytD) or 50 mM potassium chloride (KCl) as indicated. The cells were washed and incubated for 4 hours in fresh medium with the cytochalasin D or potassium chloride added back as appropriate. IL-1ß concentrations in the supernatants were determined by ELISA. *p = 0.0012, **p = 0.0005, n = 3 per experimental group.
(PDF)

S4 Fig Full-length, uncropped blots corresponding to Fig 4B. The caspase 1 p10 band corresponding to that shown in Fig 4B is indicated with the arrowhead.
(PDF)

S5 Fig Requirement for contact between bacteria and macrophages for induction of IL-1ß secretion. **A**. *B*. *infantis* or *B*. *fragilis* was added to mouse BMDMs in the presence or absence of a separating Transwell insert (Trans) with a 0.4 micron membrane. After a 1 hour incubation, the cells were washed and incubated in fresh medium overnight, with the Transwell inserts being removed to facilitate washing and then replaced after the washing step. Cell supernatants were collected and used to determine secreted IL-1ß concentrations by ELISA. *p = 0.0014, **p = 0.0009, n = 3 per experimental group. **B.** *B*. *infantis* or *B*. *fragilis* was added to mouse BMDMs in the presence or absence of a separating Transwell insert (Trans) with a 0.4 micron membrane. After a 1 hour incubation, the cells were washed and incubated in fresh medium overnight, with the Transwell inserts being removed to facilitate washing and then replaced after the washing step. Total cellular RNA was prepared and used to determine IL-1ß mRNA levels by qRT-PCR. *p = 0.0102, **p = 0.0072, n = 3 per experimental group. **C**. *B*. *infantis* or *B*. *fragilis* was added to THP-1 macrophages in the presence or absence of a separating Transwell insert (Trans) with a 0.4 micron membrane. After a 1 hour incubation, the cells were washed and incubated in fresh medium for 4 hours, with the Transwell inserts being removed to facilitate washing and then replaced after the washing step. Cell supernatants were collected and used to determine secreted IL-1ß concentrations by ELISA. *p = 0.0033, **p = 0.0002, n = 3 per experimental group. **D.** *B*. *infantis* or *B*. *fragilis* was added to THP-1 macrophages in the presence or absence of a separating Transwell insert (Trans) with a 0.4 micron membrane. After a 1 hour incubation, the cells were washed and incubated in fresh medium for 4 hours, with the Transwell inserts being removed to facilitate washing and then replaced after the washing step. Total cellular RNA was prepared and used to determine IL-1ß mRNA levels by qRT-PCR. *p = 0.0012, **p = 0.0132, n = 3 per experimental group.
(PDF)

S1 File Individual data points used to calculate the means and standard deviations represented in all the figures. (PDF)
