# Supplementary figures and images for: Commensal Bacteria-Induced Inflammasome Activation in Mouse and Human Macrophages Is Dependent on Potassium Efflux but Does Not Require Phagocytosis or Bacterial Viability
Source: PLoS One. 2016 Aug 9;11(8):e0160937. doi: 10.1371/journal.pone.0160937 (PMC4978417; doi:10.1371/journal.pone.0160937)

## Figure S1

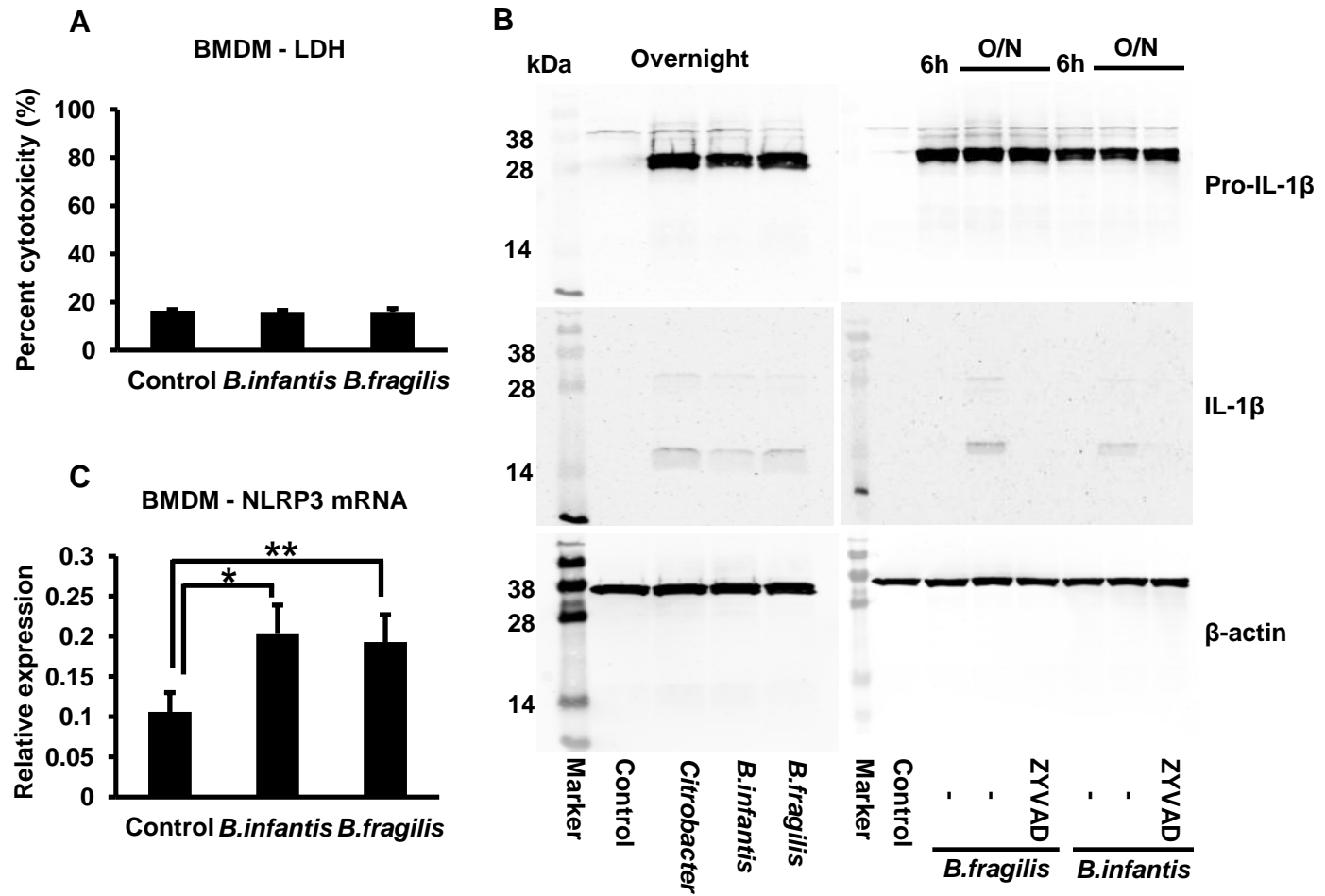

Supplement: S1 Fig — A. Mouse BMDMs were infected with B. infantis and B. fragilis for 1 hour. The cells were washed and incubated overnight in fresh medium. Cytotoxicity was determined based on the proportion of cellular LDH released. n = 3 per experimental group. B. The full-length, uncropped blots corresponding to Fig 1B. C. Mouse BMDMs were infected with B. infantis and B. fragilis for 1 hour. The cells were washed and incubated overnight in fresh medium. Total RNA was prepared and NLRP3 expression was determined by qRT-PCR. *p = 0.0203, **p = 0.0276, n = 3 per experimental group. (PDF) [file pone.0160937.s001.pdf]

Figure S2

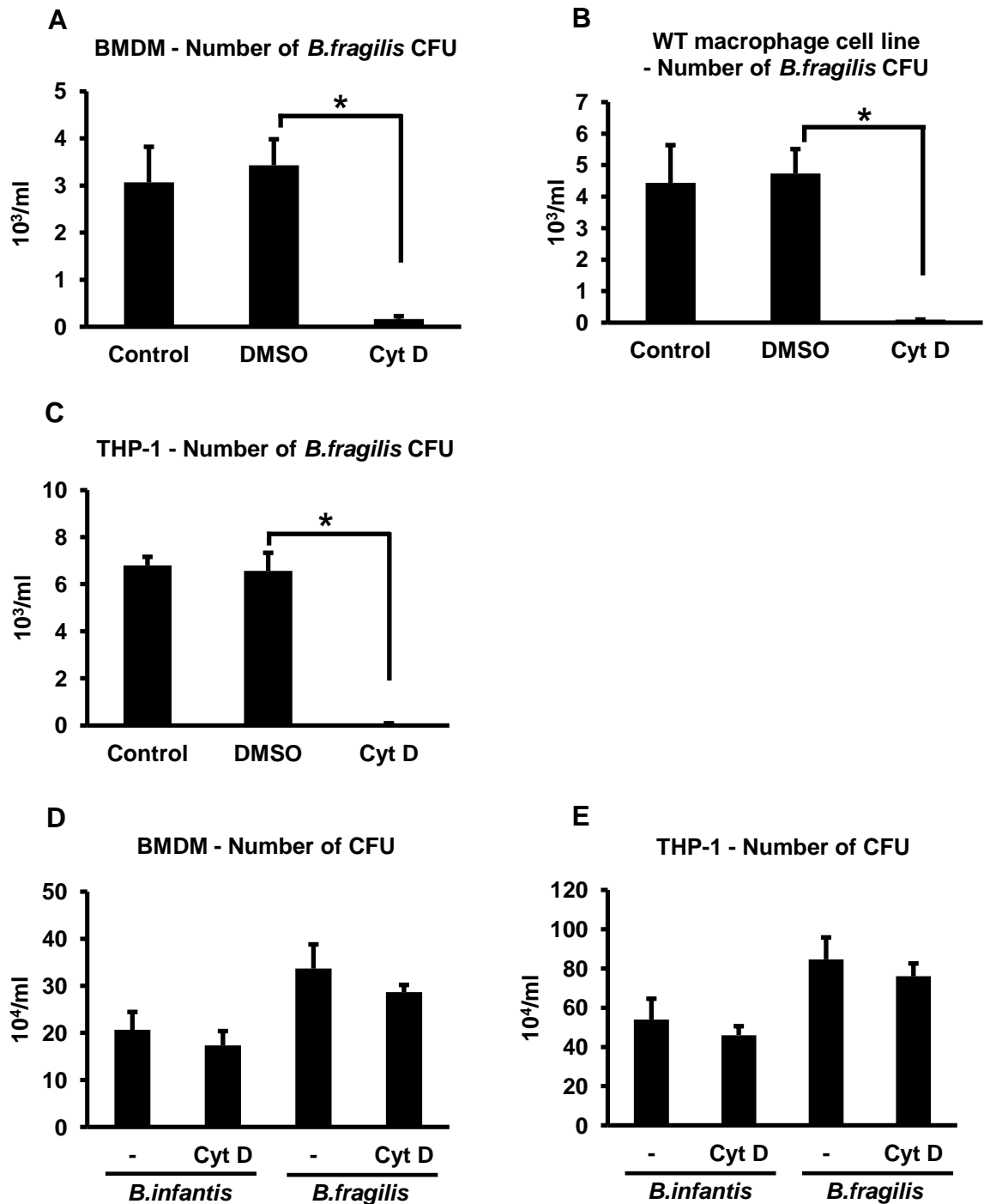

Supplement: S2 Fig — A. Mouse BMDMs were infected with B. fragilis for 1 hour in the presence of 5 μM of cytochalasin D (CytD) or an equivalent volume of DMSO, as indicated. The cells were washed and incubated for 1 hour in the presence of 200 μg/ml of gentamicin, with cytochalasin D or DMSO added back as appropriate. The cells were washed again, lysed and serial dilutions of the lysates plated to determine the numbers (colony forming units, CFU) of surviving B. fragilis. *p = 0.0088, n = 3 per experimental group. B. The WT immortalized macrophage cell line was infected with B. fragilis for 1 hour in the presence of 5 μM of cytochalasin D (CytD) or an equivalent volume of DMSO, as indicated. The cells were washed and incubated for 1 hour in the presence of 200 μg/ml of gentamicin, with cytochalasin D or DMSO added back as appropriate. The cells were washed again, lysed and serial dilutions of the lysates plated to determine the numbers (colony forming units, CFU) of surviving B. fragilis. *p = 0.0092, n = 3 per experimental group. C. THP-1 macrophages were infected with B. fragilis for 1 hour in the presence of 5 μM of cytochalasin D (CytD) or an equivalent volume of DMSO, as indicated. The cells were washed and incubated for 1 hour in the presence of 200 μg/ml of gentamicin, with cytochalasin D or DMSO added back as appropriate. The cells were washed again, lysed and serial dilutions of the lysates plated to determine the numbers (colony forming units, CFU) of surviving B. fragilis. *p = 0.0043, n = 3 per experimental group. D. Mouse BMDMs were infected with B. infantis or B. fragilis in the presence or absence of 5 μM of cytochalasin D (CytD) as indicated. The cells were washed, lysed and serial dilutions of the lysates plated to determine the numbers (colony forming units, CFU) of surviving B. infantis and B. fragilis. n = 3 per experimental group. E. THP-1 macrophages were infected with B. infantis or B. fragilis in the presence or absence of 5 μM of cytochalasin D (CytD) as indicat [file pone.0160937.s002.pdf]

Figure S3

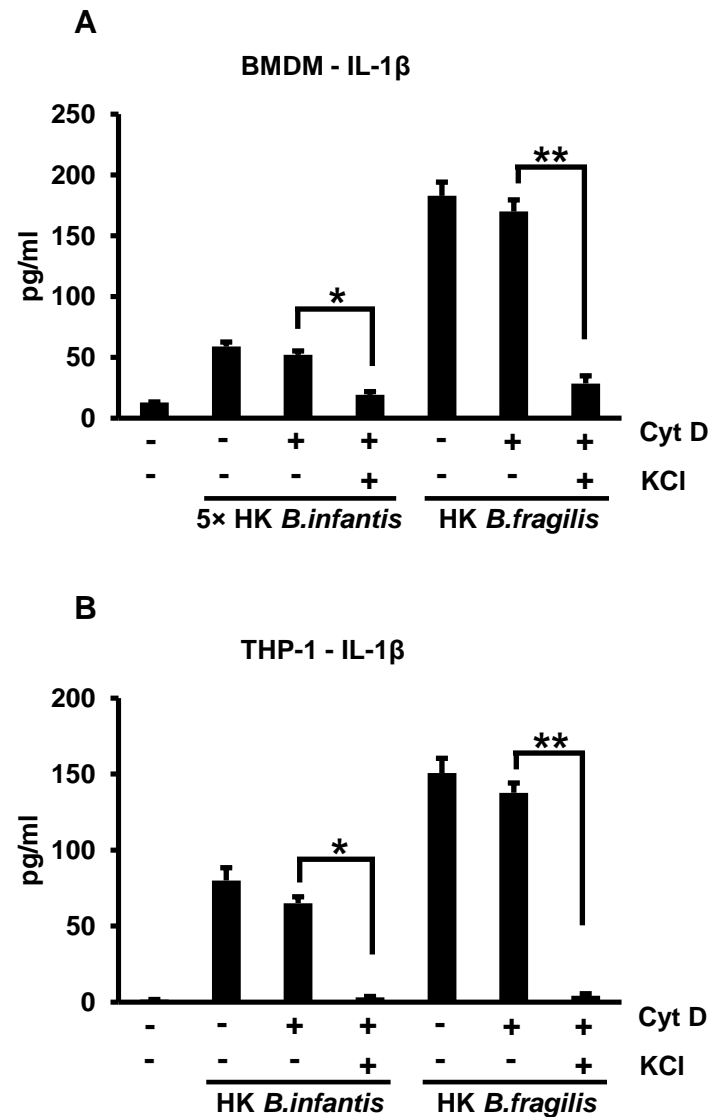

Supplement: S3 Fig — A. Mouse BMDMs were exposed to heat-killed (HK) B. infantis or B. fragilis for 1 hour in the presence of 5 μM cytochalasin D (CytD) or 50 mM potassium chloride (KCl) as indicated. The cells were washed and incubated overnight in fresh medium with the cytochalasin D or potassium chloride added back as appropriate. IL-1ß concentrations in the supernatants were determined by ELISA. *p = 0.0002, **p = 0.0001, n = 3 per experimental group. B. THP-1 macrophages were exposed to heat-killed (HK) B. infantis or B. fragilis for 1 hour in the presence of 5 μM cytochalasin D (CytD) or 50 mM potassium chloride (KCl) as indicated. The cells were washed and incubated for 4 hours in fresh medium with the cytochalasin D or potassium chloride added back as appropriate. IL-1ß concentrations in the supernatants were determined by ELISA. *p = 0.0012, **p = 0.0005, n = 3 per experimental group. (PDF) [file pone.0160937.s003.pdf]

## Figure S4

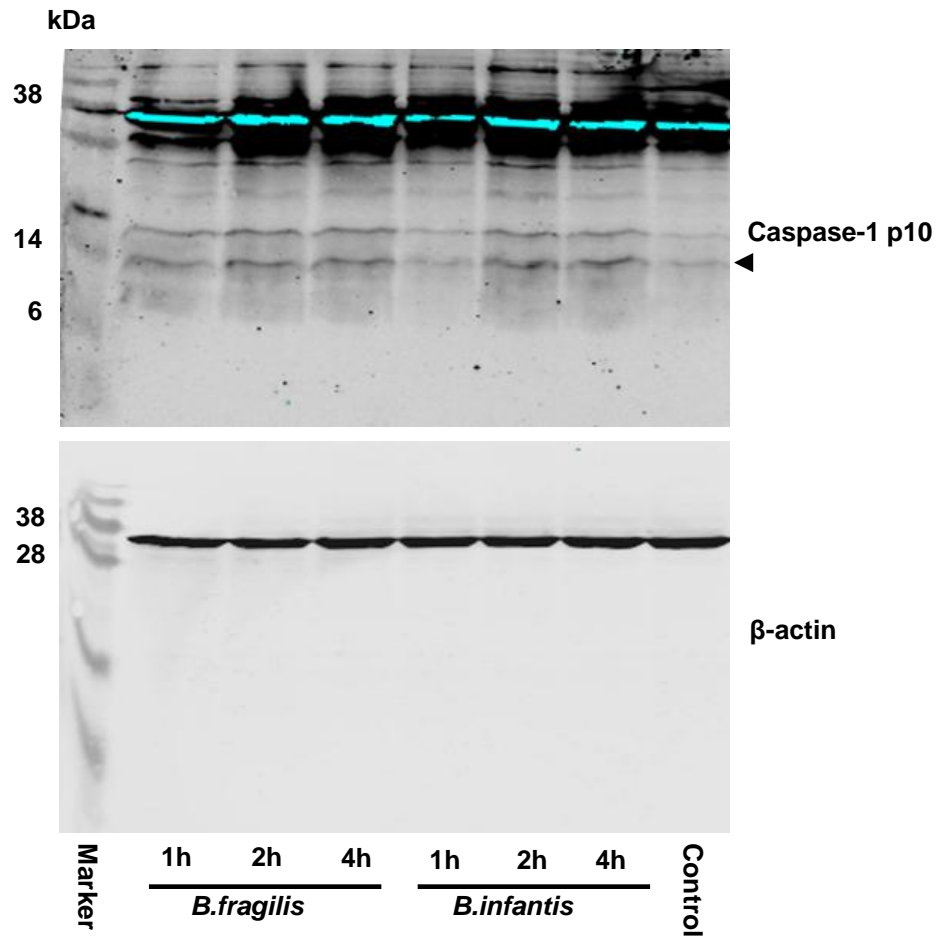

Supplement: S4 Fig — The caspase 1 p10 band corresponding to that shown in Fig 4B is indicated with the arrowhead. (PDF) [file pone.0160937.s004.pdf]

Figure S5

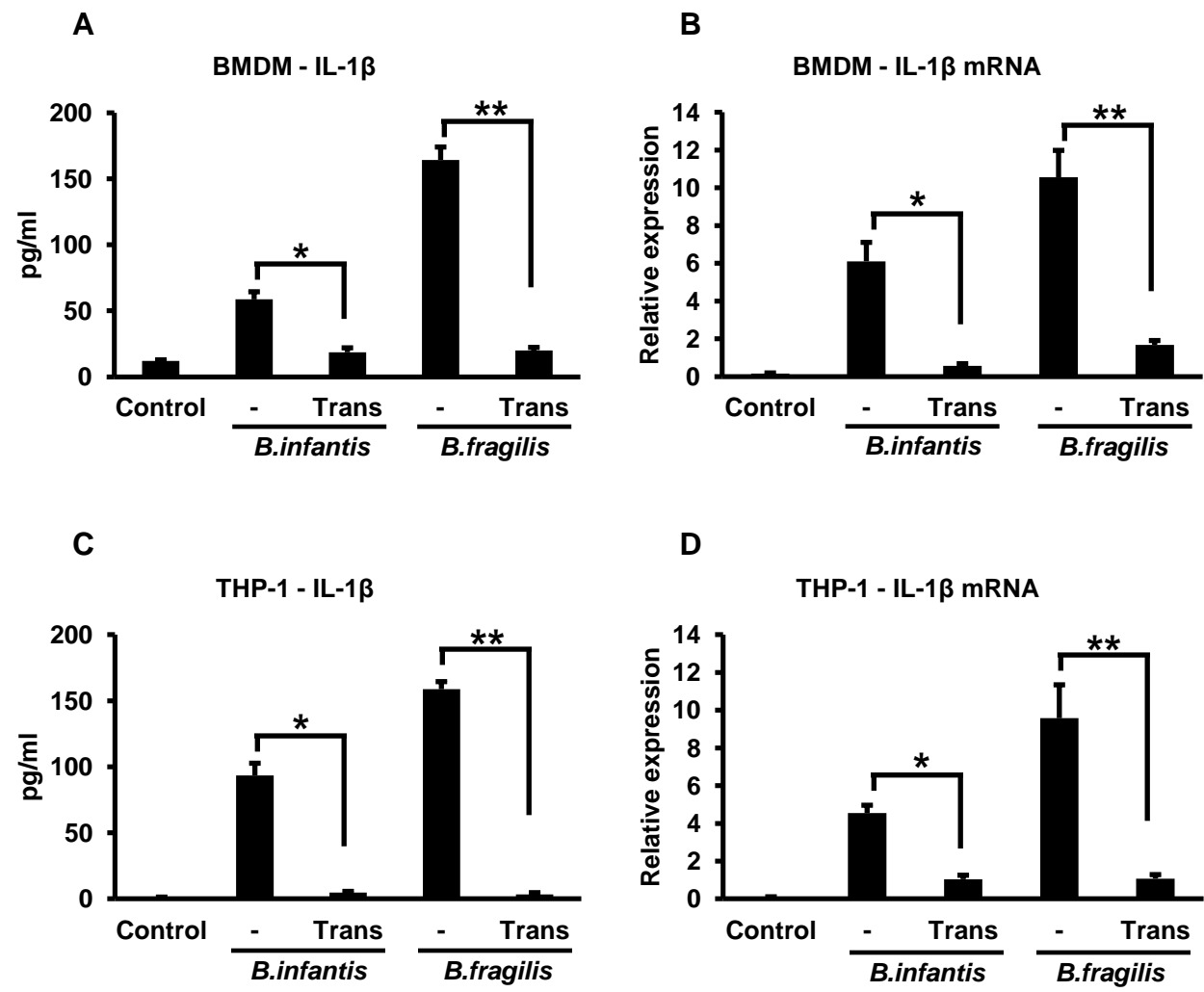

Supplement: S5 Fig — A. B. infantis or B. fragilis was added to mouse BMDMs in the presence or absence of a separating Transwell insert (Trans) with a 0.4 micron membrane. After a 1 hour incubation, the cells were washed and incubated in fresh medium overnight, with the Transwell inserts being removed to facilitate washing and then replaced after the washing step. Cell supernatants were collected and used to determine secreted IL-1ß concentrations by ELISA. *p = 0.0014, **p = 0.0009, n = 3 per experimental group. B. B. infantis or B. fragilis was added to mouse BMDMs in the presence or absence of a separating Transwell insert (Trans) with a 0.4 micron membrane. After a 1 hour incubation, the cells were washed and incubated in fresh medium overnight, with the Transwell inserts being removed to facilitate washing and then replaced after the washing step. Total cellular RNA was prepared and used to determine IL-1ß mRNA levels by qRT-PCR. *p = 0.0102, **p = 0.0072, n = 3 per experimental group. C. B. infantis or B. fragilis was added to THP-1 macrophages in the presence or absence of a separating Transwell insert (Trans) with a 0.4 micron membrane. After a 1 hour incubation, the cells were washed and incubated in fresh medium for 4 hours, with the Transwell inserts being removed to facilitate washing and then replaced after the washing step. Cell supernatants were collected and used to determine secreted IL-1ß concentrations by ELISA. *p = 0.0033, **p = 0.0002, n = 3 per experimental group. D. B. infantis or B. fragilis was added to THP-1 macrophages in the presence or absence of a separating Transwell insert (Trans) with a 0.4 micron membrane. After a 1 hour incubation, the cells were washed and incubated in fresh medium for 4 hours, with the Transwell inserts being removed to facilitate washing and then replaced after the washing step. Total cellular RNA was prepared and used to determine IL-1ß mRNA levels by qRT-PCR. *p = 0.0012, **p = 0.0132, n = 3 per experimental group. (PDF) [file pone.0160937.s005.pdf]
