# Supplementary material for: Commensal Bacteria-Induced Inflammasome Activation in Mouse and Human Macrophages Is Dependent on Potassium Efflux but Does Not Require Phagocytosis or Bacterial Viability
Source: PLoS One. 2016 Aug 9;11(8):e0160937. doi: 10.1371/journal.pone.0160937 (PMC4978417; doi:10.1371/journal.pone.0160937)
Supplement: S1 File — (PDF) [file pone.0160937.s006.pdf]

Table A<br>Data for Fig. 1, panel A				
BMDM - IL-1β (pg/ml)				
	Expt. 1	Expt. 2	Mean	SD
Control	10.177	12.155	11.521	0.959
	11.365	11.232		
	11.202	12.998		
B.infantis	53.213	88.155	71.224	16.281
	58.332	90.155		
	59.327	78.165		
B.fragilis	125.116	155.170	135.493	14.660
	112.879	138.261		
	139.164	142.365		
Citrobacter	617.065	762.167	686.132	105.436
	695.355	587.347		
	601.379	853.479		

Table B<br>Data for Fig. 1, panel C				
BMDM - IL-1β mRNA (relative expression)				
	Expt. 1	Expt. 2	Mean	SD
Control	0.036	0.075	0.049	0.018
	0.050	0.065		
	0.026	0.040		
B.infantis	2.650	3.813	3.339	0.930
	2.056	4.133		
	2.950	4.433		
B.fragilis	7.800	8.479	7.121	1.423
	5.490	7.650		
	5.165	8.145		

Table C<br>Data for Fig. 1, panel D					
BMDM - IL-1β (pg/ml)					
		Expt. 1	Expt. 2	Mean	SD
	Control	12.655	10.265	11.577	0.861
		12.366	11.265		
		11.260	11.650		
	ATP	11.265	11.265	11.657	0.632
		12.036	10.979		
		12.698	11.697		
B.infantis	-	13.326	12.365	13.447	1.172
		15.627	13.550		
		13.317	12.497		
	ATP	75.659	60.158	71.395	8.671
		82.322	62.649		
		77.265	70.317		
B.fragilis	-	13.247	12.015	13.332	1.738
		15.316	11.265		
		15.498	12.650		
	ATP	79.165	70.150	72.385	8.344
		83.165	65.498		
		75.165	61.170		

Table D<br>Data for Fig. 2, panel A					
BMDM - IL-1β (pg/ml)					
		Expt. 1	Expt. 2	Mean	SD
	Control	12.531	12.655	12.597	0.865
		11.488	13.655		
		11.794	13.457		
B.infantis	-	53.730	80.655	69.875	12.272
		57.269	75.101		
		68.880	83.619		
	HK	13.654	15.621	14.645	1.249
		14.003	16.216		
		13.056	15.321		
	5× HK	95.312	72.316	84.776	14.925
		75.332	105.322		
		68.060	92.316		

Table E<br>Data for Fig. 2, panel B					
BMDM - IL-1β (pg/ml)					
		Expt. 1	Expt. 2	Mean	SD
	Control	11.641	12.648	12.750	1.177
		14.455	11.245		
		13.266	13.246		
	DMSO	12.346	13.216	12.846	0.905
		13.346	14.216		
		12.158	11.794		
B.infantis	-	58.655	43.265	57.776	8.102
		67.013	58.165		
		56.360	63.197		
	Cyt D	39.032	46.123	48.655	7.102
		54.415	55.014		
		42.365	54.980		
B.fragilis	-	155.797	170.457	165.742	16.489
		179.792	142.548		
		187.063	158.794		
	Cyt D	152.370	134.579	150.479	11.619
		161.329	149.308		
		140.599	164.688		
	HK	171.715	193.785	178.879	10.130
		180.146	164.365		
		184.097	179.165		

Table F<br>Data for Fig. 3, panel A					
WT macrophage cell line - IL-1β (pg/ml)					
		Expt. 1	Expt. 2	Mean	SD
	Control	12.659	12.490	12.205	0.678
		12.955	11.036		
		11.927	12.165		
B.infantis	-	42.322	62.155	58.224	10.376
		58.317	70.691		
		50.370	65.490		
	Cyt D	36.370	55.015	45.000	11.816
		39.217	45.113		
		31.642	62.646		
	HK	13.337	12.013	13.098	1.096
		14.356	11.895		
		14.336	12.650		
	5× HK	65.216	70.570	65.146	7.565
		53.322	75.216		
		65.336	61.214		
B.fragilis	-	157.667	120.116	135.910	12.721
		130.659	130.550		
		141.326	135.144		
	Cyt D	90.216	100.135	82.142	12.607
		62.978	78.546		
		77.322	83.655		
	HK	165.316	189.264	158.915	21.833
		155.326	135.346		
		134.032	174.203		

Table G<br>Data for Fig. 3, panel B					
Mouse macrophage cell line - IL-1β (pg/ml)					
		Expt. 1	Expt. 2	Mean	SD
Control	WT	12.197	12.550	12.369	0.467
		11.759	13.165		
		12.227	12.316		
	NLRP3 KO	16.235	12.050	13.709	1.695
		15.249	13.499		
		13.056	12.165		
B.infantis	WT	56.066	47.166	50.886	4.127
		52.565	45.317		
		54.065	50.135		
	NLRP3 KO	26.364	20.912	22.324	4.920
		26.759	18.458		
		26.337	15.114		
B.fragilis	WT	142.323	92.655	112.916	21.751
		118.355	89.216		
		132.599	102.350		
	NLRP3 KO	30.266	22.133	27.055	5.085
		31.125	25.843		
		32.649	20.316		

Table H<br>Data for Fig. 3, panel C					
WT macrophage cell line - IL-1β (pg/ml)					
		Expt. 1	Expt. 2	Mean	SD
	Control	13.065	12.013	12.600	0.978
		13.557	12.498		
		13.464	11.003		
	KCl	12.926	11.791	12.373	0.516
		11.850	12.113		
		12.688	12.871		
	RR	14.660	12.047	13.202	1.038
		12.659	12.302		
		13.479	14.068		
B.infantis	-	64.927	89.216	72.274	15.383
		61.360	90.316		
		52.562	75.261		
	KCl	22.817	20.316	19.058	3.771
		20.480	15.116		
		21.975	13.644		
	RR	31.791	35.650	33.307	4.784
		30.149	30.590		
		29.644	42.016		
B.fragilis	-	124.365	105.315	117.266	14.085
		129.971	112.750		
		133.080	98.113		
	KCl	28.432	15.146	23.832	5.528
		29.615	20.136		
		26.715	22.946		
	RR	54.015	35.948	40.903	7.844
		42.215	30.590		
		42.485	40.162		

Table I<br>Data for Fig. 4, panel A					
THP-1 - IL-1β (pg/ml)					
		Expt. 1	Expt. 2	Mean	SD
	Control	2.218	1.549	2.431	0.874
		2.530	2.040		
		4.092	2.155		
	DMSO	2.017	2.050	2.080	0.723
		3.216	1.479		
		2.515	1.204		
B.infantis	-	108.897	85.147	97.731	9.096
		107.318	92.364		
		98.056	94.602		
	50μM ZYVAD	52.906	48.922	52.659	5.288
		62.709	50.362		
		52.807	48.246		
	100μM ZYVAD	35.882	25.150	26.245	5.260
		26.699	20.365		
		26.208	23.165		
Citrobacter		189.657	158.497	163.242	20.439
		165.468	140.265		
		183.216	142.346		

<table>
<tr><th colspan="6">Table J
Data for Fig. 5, panel A</th></tr>
<tr><th colspan="6">THP-1 - IL-1β (pg/ml)</th></tr>
<tr><th></th><th></th><th>Expt. 1</th><th>Expt. 2</th><th>Mean</th><th>SD</th></tr>
<tr><td rowspan="6"></td><td rowspan="3">Control</td><td>0.613</td><td>1.855</td><td rowspan="3">1.326</td><td rowspan="3">0.846</td></tr>
<tr><td>0.549</td><td>2.146</td></tr>
<tr><td>0.529</td><td>2.265</td></tr>
<tr><td rowspan="3">KCl</td><td>0.648</td><td>1.857</td><td rowspan="3">1.334</td><td rowspan="3">0.789</td></tr>
<tr><td>0.688</td><td>2.326</td></tr>
<tr><td>0.554</td><td>1.933</td></tr>
<tr><td rowspan="6">B.infantis</td><td rowspan="3">-</td><td>93.654</td><td>110.349</td><td rowspan="3">98.234</td><td rowspan="3">14.001</td></tr>
<tr><td>85.364</td><td>87.022</td></tr>
<tr><td>92.649</td><td>120.365</td></tr>
<tr><td rowspan="3">KCl</td><td>8.491</td><td>10.241</td><td rowspan="3">9.487</td><td rowspan="3">0.721</td></tr>
<tr><td>9.653</td><td>9.442</td></tr>
<tr><td>8.841</td><td>10.255</td></tr>
<tr><td colspan="6"></td></tr>
<tr><th colspan="6">THP-1 - IL-1β (pg/ml)</th></tr>
<tr><th></th><th></th><th>Expt. 1</th><th>Expt. 2</th><th>Mean</th><th>SD</th></tr>
<tr><td rowspan="6"></td><td rowspan="3">Control</td><td>2.315</td><td>2.365</td><td rowspan="3">1.828</td><td rowspan="3">0.446</td></tr>
<tr><td>1.259</td><td>1.890</td></tr>
<tr><td>1.629</td><td>1.509</td></tr>
<tr><td rowspan="3">RR</td><td>1.563</td><td>2.294</td><td rowspan="3">1.891</td><td rowspan="3">0.386</td></tr>
<tr><td>1.512</td><td>2.365</td></tr>
<tr><td>1.593</td><td>2.020</td></tr>
<tr><td rowspan="6">B.infantis</td><td rowspan="3">-</td><td>87.217</td><td>102.658</td><td rowspan="3">95.073</td><td rowspan="3">10.945</td></tr>
<tr><td>79.302</td><td>110.255</td></tr>
<tr><td>95.698</td><td>95.310</td></tr>
<tr><td rowspan="3">RR</td><td>33.456</td><td>40.361</td><td rowspan="3">37.071</td><td rowspan="3">5.049</td></tr>
<tr><td>32.155</td><td>45.022</td></tr>
<tr><td>33.215</td><td>38.216</td></tr>
</table>

Table K<br>Data for Fig. 5, panel B					
THP-1 - IL-1β (pg/ml)					
		Expt. 1	Expt. 2	Mean	SD
	Control	0.642	2.032	1.305	0.605
		0.872	1.853		
		0.792	1.642		
	Apyrase	0.922	1.236	1.119	0.415
		0.872	1.665		
		0.548	1.471		
B.infantis	-	86.583	105.216	95.271	14.877
		79.581	120.340		
		90.689	89.216		
	Apyrase	82.060	123.655	94.298	18.908
		78.427	92.316		
		78.540	110.788		

Table L<br>Data for Fig. 5, panel C					
THP-1 - IL-1β (pg/ml)					
		Expt. 1	Expt. 2	Mean	SD
	Control	0.721	0.892	0.710	0.111
		0.681	0.597		
		0.600	0.766		
B.infantis	-	82.655	92.655	93.096	17.436
		81.365	122.155		
		75.589	104.156		
	HK	120.026	129.216	110.099	16.414
		115.669	104.018		
		81.549	110.117		
	Cyt D	74.017	85.312	81.049	7.951
		72.622	90.655		
		88.320	75.366		

Table M<br>Data for Fig. 5, panel D					
THP-1 - IL-1β (pg/ml)					
		Expt. 1	Expt. 2	Mean	SD
	Control	1.326	2.015	1.543	0.522
		1.168	2.216		
		0.850	1.680		
B.fragilis	-	128.729	146.014	138.167	11.870
		127.649	153.942		
		126.649	146.019		
	HK	167.317	145.603	164.118	19.742
		172.265	174.017		
		136.036	189.472		
	Cyt D	118.479	134.015	115.319	14.425
		105.051	100.255		
		130.145	103.972		
	KCl	7.562	7.258	8.160	0.836
		7.730	9.155		
		8.032	9.225		
	Apyrase	147.016	158.032	133.141	17.276
		130.650	120.489		
		132.165	110.493		

Table N<br>Data for Fig. 6, panel A					
Human MDM - IL-1β (pg/ml)					
		Expt. 1	Expt. 2	Mean	SD
	Control	0.019	0.022	0.055	0.086
		0.021	0.016		
		0.021	0.231		
B.infantis	-	3.903	4.671	3.839	0.549
		3.216	3.482		
		3.497	4.264		
	HK	3.916	4.021	4.148	0.761
		3.288	5.565		
		3.879	4.216		
	Cyt D	3.022	3.249	3.291	0.500
		4.280	3.063		
		2.916	3.216		
	KCl	0.030	0.045	0.031	0.011
		0.023	0.024		
		0.021	0.045		

Table O<br>Data for Fig. 6, panel B					
Human MDM - IL-1β (pg/ml)					
		Expt. 1	Expt. 2	Mean	SD
	Control	0.016	0.014	0.016	0.002
		0.016	0.020		
		0.015	0.017		
HK *B.infantis*	-	4.466	5.176	4.427	0.387
		4.132	4.313		
		4.316	4.159		
	Cyt D	3.549	4.057	3.805	0.599
		4.894	3.248		
		3.416	3.665		
	Cyt D +KCl	0.096	0.050	0.081	0.018
		0.085	0.089		
		0.070	0.093		

Table P<br>Data for Fig. 6, panel C				
**Human MDM - IL-1β mRNA (relative expression)**				
	Expt. 1	Expt. 2	Mean	SD
Control	0.073	0.054	0.055	0.015
	0.045	0.062		
	0.062	0.032		
Pam3Cys	36.367	27.015	26.895	6.845
	26.316	28.302		
	28.316	15.055		
**Human MDM - IL-1β (pg/ml)**				
	Expt. 1	Expt. 2	Mean	SD
Control	0.345	0.248	0.354	0.087
	0.342	0.515		
	0.325	0.348		
Pam3Cys	0.413	0.492	0.432	0.080
	0.523	0.481		
	0.321	0.361		

Table Q<br>Data for Fig. S1, panel A			
BMDM - LDH (%)			
		Mean	SD
Control	16.654	16.459	0.438
	16.765		
	15.957		
B.infantis	15.264	15.960	0.605
	16.361		
	16.255		
B.infantis	17.514	15.890	1.421
	15.283		
	14.875		

Table R<br>Data for Fig. S1, panel C			
BMDM - NLRP3 mRNA (relative expression)			
		Mean	SD
Control	0.089	0.106	0.024
	0.096		
	0.133		
B.infantis	0.170	0.204	0.035
	0.240		
	0.203		
B.fragilis	0.212	0.193	0.034
	0.213		
	0.153		

Table S<br>Data for Fig. S2, panel A			
BMDM - Number of *B.fragilis* CFU (10^3/ml)			
		Mean	SD
Control	3.6	3.067	0.757
	2.2		
	3.4		
DMSO	2.9	3.433	0.551
	3.4		
	4.0		
Cyt D	0.2	0.167	0.058
	0.2		
	0.1		

Table T<br>Data for Fig. S2, panel B			
WT macrophage cell line<br>- Number of *B.fragilis* CFU (10^3/ml)			
		Mean	SD
Control	4.5	4.433	1.201
	5.6		
	3.2		
DMSO	4.1	4.733	0.777
	4.5		
	5.6		
Cyt D	0.1	0.100	0.000
	0.1		
	0.1		

Table U<br>Data for Fig. S2, panel C			
THP-1 - Number of *B.fragilis* CFU (10^3/ml)			
		Mean	SD
Control	7.1	6.800	0.361
	6.9		
	6.4		
DMSO	7.4	6.567	0.764
	6.4		
	5.9		
Cyt D	0	0.033	0.058
	0		
	0.1		

Table V<br>Data for Fig. S2, panel D				
BMDM - Number of CFU (10^4/ml)				
			Mean	SD
B.infantis	-	18	20.667	3.786
		19		
		25		
	Cyt D	20	17.333	3.055
		18		
		14		
B.fragilis	-	35	33.667	5.132
		38		
		28		
	Cyt D	29	28.667	1.528
		27		
		30		

Table W<br>Data for Fig. S2, panel E				
THP-1 - Number of CFU (10^4/ml)				
			**Mean**	**SD**
B.infantis	**-**	**58**	**54.000**	**10.583**
		**42**		
		**62**		
	**Cyt D**	**50**	**46.000**	**4.583**
		**41**		
		**47**		
B.fragilis	**-**	**89**	**84.667**	**11.150**
		**93**		
		**72**		
	**Cyt D**	**82**	**76.000**	**6.557**
		**69**		
		**77**		

Table X<br>Data for Fig. S3, panel A				
BMDM - IL-1β (pg/ml)				
			Mean	SD
	Control	13.233	12.902	0.378
		12.490		
		12.984		
5× HK *B.infantis*	-	59.116	58.790	3.748
		54.890		
		62.365		
	Cyt D	48.699	51.989	3.236
		52.100		
		55.168		
	Cyt D +KCl	22.032	19.315	2.452
		18.650		
		17.264		
HK *B.fragilis*	-	189.166	182.697	11.291
		169.660		
		189.265		
	Cyt D	170.156	169.802	9.597
		179.216		
		160.033		
	Cyt D +KCl	35.321	28.494	6.287
		27.216		
		22.944		

Table Y<br>Data for Fig. S3, panle B				
THP-1 - IL-1β (pg/ml)				
			Mean	SD
	Control	1.747	1.609	0.120
		1.551		
		1.529		
HK *B.infantis*	-	86.365	80.014	8.380
		70.516		
		83.160		
	Cyt D	67.916	65.099	4.164
		67.065		
		60.316		
	Cyt D +KCl	2.615	3.134	0.586
		3.769		
		3.017		
HK *B.fragilis*	-	157.316	150.560	9.778
		139.348		
		155.015		
	Cyt D	140.361	137.676	6.419
		142.316		
		130.350		
	Cyt D +KCl	5.615	4.283	1.222
		4.022		
		3.212		

Table Z<br>Data for Fig. S5, panel A				
BMDM - IL-1β (pg/ml)				
			Mean	SD
	Control	11.335	12.156	0.714
		12.635		
		12.498		
B.infantis	-	64.650	58.660	5.758
		53.165		
		58.165		
	Transwell	15.498	18.633	3.351
		18.237		
		22.165		
B.fragilis	-	175.497	164.266	9.740
		159.165		
		158.136		
	Transwell	20.149	19.967	2.334
		22.205		
		17.547		

Table AA<br>Data for Fig. S5, panel B				
BMDM - IL-1β mRNA (relative expression)				
			Mean	SD
	Control	0.126	0.160	0.030
		0.169		
		0.185		
B.infantis	-	5.979	6.105	1.006
		7.168		
		5.168		
	Transwell	0.692	0.573	0.108
		0.546		
		0.480		
B.fragilis	-	12.015	10.559	1.425
		10.493		
		9.168		
	Transwell	1.595	1.670	0.237
		1.480		
		1.935		

Table BB<br>Data for Fig. S5, panel C				
THP-1 - IL-1β (pg/ml)				
			Mean	SD
	Control	1.235	1.249	0.092
		1.165		
		1.347		
B.infantis	-	102.906	93.452	9.225
		84.474		
		92.976		
	Transwell	3.943	4.662	0.996
		5.798		
		4.243		
B.fragilis	-	164.915	158.736	5.728
		153.602		
		157.690		
	Transwell	3.177	3.369	1.321
		2.155		
		4.776		

Table CC<br>Data for Fig. S5, panel D				
THP-1 - IL-1β mRNA (relative expression)				
			Mean	SD
	Control	0.103	0.097	0.012
		0.084		
		0.105		
B.infantis	-	4.847	4.542	0.419
		4.064		
		4.714		
	Transwell	0.851	1.043	0.201
		1.253		
		1.026		
B.fragilis	-	9.747	9.575	1.768
		11.251		
		7.727		
	Transwell	0.928	1.076	0.209
		0.984		
		1.315		
